# Age-related differences in corticospinal and reticulospinal adaptations to short-term strength training

**DOI:** 10.1007/s00421-026-06222-9

**Published:** 2026-04-10

**Authors:** Ummatul Siddique, Ashlyn K. Frazer, Jamie Tallent, Oliver Hayman, Yonas Akalu, Mohamad Rostami, Sergio Uribe, Simon Walker, Dawson J. Kidgell

**Affiliations:** 1 Monash University Exercise Neuroplasticity Research Unit, School of Primary and Allied Care, Monash University, Frankston, Australia, Monash University, PO Box 527, Frankston, Australia; 2 School of Sport, Rehabilitation and Exercise Sciences, University of Essex, Colchester, UK; 3 School of Cardiovascular and Metabolic Health, College of Medical, Veterinary and Life Sciences, University of Glasgow, Glasgow, UK; 4 Department of Human Physiology, School of Medicine, University of Gondar, Gondar, Ethiopia; 5 Department of Medical Imaging and Radiation Sciences, School of Primary and Allied Care, Monash University, Clayton, Australia; 6 Faculty of Sport and Health Sciences, NeuroMuscular Research Center, University of Jyväskylä, Jyväskylä, Finland

**Keywords:** Reticulospinal tract, Corticospinal excitability, Ageing, Rate of force development, Motor training

## Abstract

**Purpose:**

High force production depends on effective transmission of motor commands to spinal motoneurones, a capacity that declines with age. Although strength adaptations are often attributed to cortical mechanisms, subcortical pathways providing strong descending drive remain understudied. The reticulospinal tract, with its fast, widespread projections to motoneurones, is well positioned to support force generation, yet its role in human strength adaptation is largely unknown. This study examined age-related differences in corticospinal and reticulospinal adaptations following short-term strength training.

**Methods:**

Thirteen older adults (67 ± 5 years) and twelve younger adults (26 ± 6 years) completed a two-week unilateral elbow-flexor strength training programme. Neurophysiological and behavioural assessments were performed to evaluate cortical and subcortical activity and their contribution to strength gains.

**Results:**

Both groups demonstrated similar increases in strength; however, corticospinal excitability decreased in younger adults and remained unchanged in older adults. Reduced GABAergic inhibition, indexed by shortened cortical silent periods, occurred in both groups. Reaction time and rate of force development in response to startling stimuli improved in both groups, with a more pronounced StartReact effect in older adults, consistent with enhanced reticulospinal responsiveness. Voluntary drive, measured by central activation ratio, increased significantly in older adults (*p* < 0.001), whereas M_MAX_ remained stable.

**Conclusion:**

Early strength gains were accompanied by reduced intracortical inhibition and increased engagement of subcortical descending pathways. The pronounced StartReact responses and enhanced early-force kinetics provide indirect evidence of greater reticulospinal contribution, with older adults appearing to rely more heavily on this pathway when corticospinal plasticity is limited.

**Supplementary Information:**

The online version contains supplementary material available at 10.1007/s00421-026-06222-9.

## Highlights

Short-term strength training improved rate of force development and reaction time in older and younger adults.

Corticospinal excitability decreased in younger adults after training, with no change in older adults, indicating age-related differences in corticospinal plasticity.

GABAergic inhibition, as indexed by cortical silent period (cSP), was reduced in both groups following training.

**Supplementary Information:**

The online version contains supplementary material available at 10.1007/s00421-026-06222-9.

##  Introduction

Age-related declines in muscle strength, typically ranging from 20 to 40% by the seventh to eighth decade, are commonly attributed to degenerative changes in the central nervous system (Doherty 2003; Hunter et al. 2016). These declines are clinically significant, undermining functional autonomy and reducing quality of life in older adults (Manini et al. 2007; Rantanen et al. 1999; Visser et al. 2000). While neural degeneration is closely linked to motor impairment (Clark and Taylor 2011), the precise neurophysiological mechanisms driving this strength loss remain incompletely defined. Strength training is a well-established countermeasure to age-related deficits in force production (Caserotti et al. 2008; Häkkinen et al. 2000, 2001). Early strength gains (≤ 4 weeks) are primarily mediated by neural rather than morphological adaptations (Carroll et al. 2002; Moritani and DeVries 1979; Sale 1988). In young adults, these neural adaptations are marked by increased corticospinal excitability (CSE) and reduced intracortical inhibition (Hendy and Kidgell 2013; Kidgell et al. 2010; Leung et al. 2017; Mason et al. 2020; Pearce et al. 2013), implicating the corticospinal tract (CST) as a key conduit for motor plasticity during early training phases (Kidgell et al. 2017; Siddique et al. 2020).

Despite the well-documented functional benefits of strength training in older adults (Caserotti et al. 2008; Häkkinen et al. 2000, 2001; Marques et al. 2023), relatively few studies have systematically examined whether the ageing brain retains the capacity for corticospinal plasticity (Siddique et al. 2022). This underscores a significant gap in the literature, particularly in light of consistent cross-sectional evidence demonstrating age-related reductions in CSE and modifications in intracortical inhibitory function (Hortobagyi et al. 2001; Oliviero et al. 2006; Sale and Semmler 2005), neurophysiological changes that may constrain plasticity in the ageing motor system. Although older adults exhibit measurable improvements in strength, the neurophysiological mechanisms underpinning these gains remain poorly characterised. Only three studies to date have directly assessed corticospinal adaptation following strength training in older populations (Christie and Kamen 2014; Gomez-Guerrero et al. 2024; Siddique et al. 2025). Christie and Kamen (2014) reported a reduction in corticospinal inhibition without concurrent changes in CSE after two weeks of dorsiflexor training. Conversely, Guerrero et al. (2024) observed improved strength yet paradoxically found decreased CSE following a seven-week whole-body strength training program. Recently, Siddique et al. (2025) reported that following a single session of strength exercise, older adults exhibited concurrent reductions in CSE and intracortical inhibition. These conflicting findings suggest age-specific neural adaptations that diverge from established models based on younger adults (Siddique et al. 2020), underscoring the need to clarify the neurophysiological constraints and plastic capacity of the ageing motor system.

While the CST has long dominated models of motor control and plasticity, growing evidence underscores the reticulospinal tract (RST) as a parallel and potentially essential driver of strength adaptation (Akalu et al. 2025; Baker 2011). Originating in the pontomedullary reticular formation, the RST projects bilaterally to spinal α-motoneurones and contributes to the control of both proximal and distal musculature (Davidson et al. 2007; Jankowska et al. 2003; Peterson et al. 1975). Non-human primate studies demonstrate that early strength gains can occur without measurable changes in CST activity, implicating the RST as a primary mediator of neural adaptation (Akalu et al. 2025; Glover and Baker 2020). In humans, however, direct evidence of RST plasticity in response to strength training remains limited (Siddique et al.^2025^; Mooney et al.^ 2025^).

Importantly, emerging work suggests that ageing constrains subcortical motor plasticity. Mooney et al. (2025) demonstrated a marked reduction in RST excitability in older adults, reflected by lower ipsilateral motor-evoked potential (iMEP) presence and reduced flexor strength. Complementing this, Siddique et al. (2025) reported that, although both young and older adults exhibited reduced short-interval cortical inhibition (SICI) and shorter silent periods (SP) following acute high-intensity strength exercise, only young adults demonstrated increased CSE. Older adults showed no enhancement of reaction time or StartReact responses, indicating limited RST responsiveness to the same training stimulus. Collectively, these findings suggest that RST plasticity is reduced at baseline and less responsive to acute strength training in older adults.

Given these acute and baseline deficits, the next question is why the ageing RST shows limited plasticity. Age-related degeneration within brainstem nuclei (Bouhrara et al. 2020; Lambert et al. 2013) provides one plausible mechanism, as reductions in monoaminergic drive and structural integrity could constrain both excitability and adaptive capacity. However, this alone does not account for the variability seen in older adults. Stronger individuals show markedly greater RST excitability than weaker peers, suggesting compensatory recruitment of this pathway when corticospinal output is compromised (Maitland and Baker 2021). Such variability points to an underappreciated reserve within the RST, albeit one expressed inconsistently across individuals. Despite its clear relevance, the RST remains poorly characterised in human neuroplasticity research; its deep anatomical location and the historical lack of non-invasive measures have limited progress (Akalu et al. 2023).

Although TMS is well suited for probing corticospinal and intracortical circuits (Barker et al. 1985; Perez and Cohen 2009), non-invasive assessment of the RST is more challenging because of its deep origin and diffuse projections. The StartReact paradigm activates the reticular formation using a startling acoustic stimulus, eliciting rapid subcortically mediated motor responses (Davis et al. 1982; Hammond 1973; Tapia et al. 2022). When paired with a visual go-cue, the stimulus triggers the involuntary release of a prepared movement and markedly shortens reaction time (Baker and Perez 2017; Carlsen et al. 2004). This response provides a functional index of RST excitability. Given the RST’s role in force generation (Akalu et al. 2023) and its proposed compensatory function when corticospinal drive declines with age (Maitland and Baker 2021), determining whether this pathway adapts to strength training is important for understanding age-related alterations in descending motor control.

This study investigated how short-term, high-intensity metronome-paced strength training (MPST) influences corticospinal and reticulospinal pathways in older adults, with direct comparison to younger individuals. MPST was selected because externally paced contractions consistently produce stronger corticospinal modulation than self-paced protocols (Gómez-Feria et al. 2023; Gordon et al. 2024; Leung et al. 2017). Gómez-Feria et al. (2023), for example, reported increased CSE and reduced SICI after MPST, effects attributed to improved temporal precision of descending drive, changes not observed with self-paced training. However, while cortical adaptations to MPST are becoming clearer, its effect on subcortical pathways remains largely unknown (Akalu et al. 2025). By assessing both corticospinal and reticulospinal responsiveness in young and older adults, this study examined whether early strength gains reflect distributed plasticity across multiple descending systems.

## Methods

### Sample size estimation and participant characteristics

A power analysis for this study was conducted using G*Power software. Corticospinal inhibition was selected as the primary outcome to estimate the required sample size. Based on a similar study by Christie and Kamen (2014), which reported a between-group effect size of f = 0.45 for pre–post changes in silent period following two weeks of strength training in older adults, we set the desired power (1–β) at 0.80 and the significance level (α) at 0.05. The power analysis for a repeated-measures, mixed-design ANOVA indicated that a minimum of 10 participants would be required. To account for potential attrition (estimated at 20%), we aimed to recruit approximately 12–13 participants per group. Ultimately, we enrolled 13 older adults and 12 young adults in the study.

Following community advertisements, 65 individuals expressed interest in participating. After screening, 25 participants were enrolled: 13 older adults (5 males, 8 females; mean ± SD: age = 67 ± 5 years, height = 166 ± 9 cm, body mass = 77 ± 15 kg, BMI = 28 ± 6 kg/m²) and 12 young adults (6 males, 6 females; age = 26 ± 6 years, height = 170 ± 10 cm, body mass = 69 ± 11 kg, BMI = 24 ± 3 kg/m²). Eligibility was assessed through comprehensive screening for any musculoskeletal disorders, neurological conditions, head trauma, implants or use of any anti-depressant. TMS Adults Safety Questionnaire (Keel et al. 2001) was used to assess the eligibility for brain stimulation and physical activity levels were screened via the Physical Activity Readiness Questionnaire (PAR-Q) and the International Physical Activity Questionnaire (IPAQ). None of the participants had engaged in strength training in the 12 months prior to the study. Hand dominance was assessed using the Edinburgh Handedness Inventory (Oldfield 1971), with mean laterality scores of 69 ± 50 for older adults and 81 ± 18 for young adults. All participants provided written informed consent before participation. The study was approved by the Monash University Human Research Ethics Committee (Project ID: 30882) and conducted in accordance with the Declaration of Helsinki.

### Experimental set up & Strength training protocol

Prior to the study, participants attended a familiarisation session in the laboratory. Anthropometric data (height and body mass) were recorded, and participants were introduced to all experimental procedures, including transcranial magnetic stimulation (TMS), peripheral nerve stimulation, and the StartReact protocol, to ensure they were comfortable with the testing protocols. Strength assessments, including one-repetition maximum (1-RM) and maximum voluntary force (MVF) of the elbow flexors, were also conducted. Both groups were familiarised with the training protocol, including synchronisation with a metronome.

**Fig. 1:**
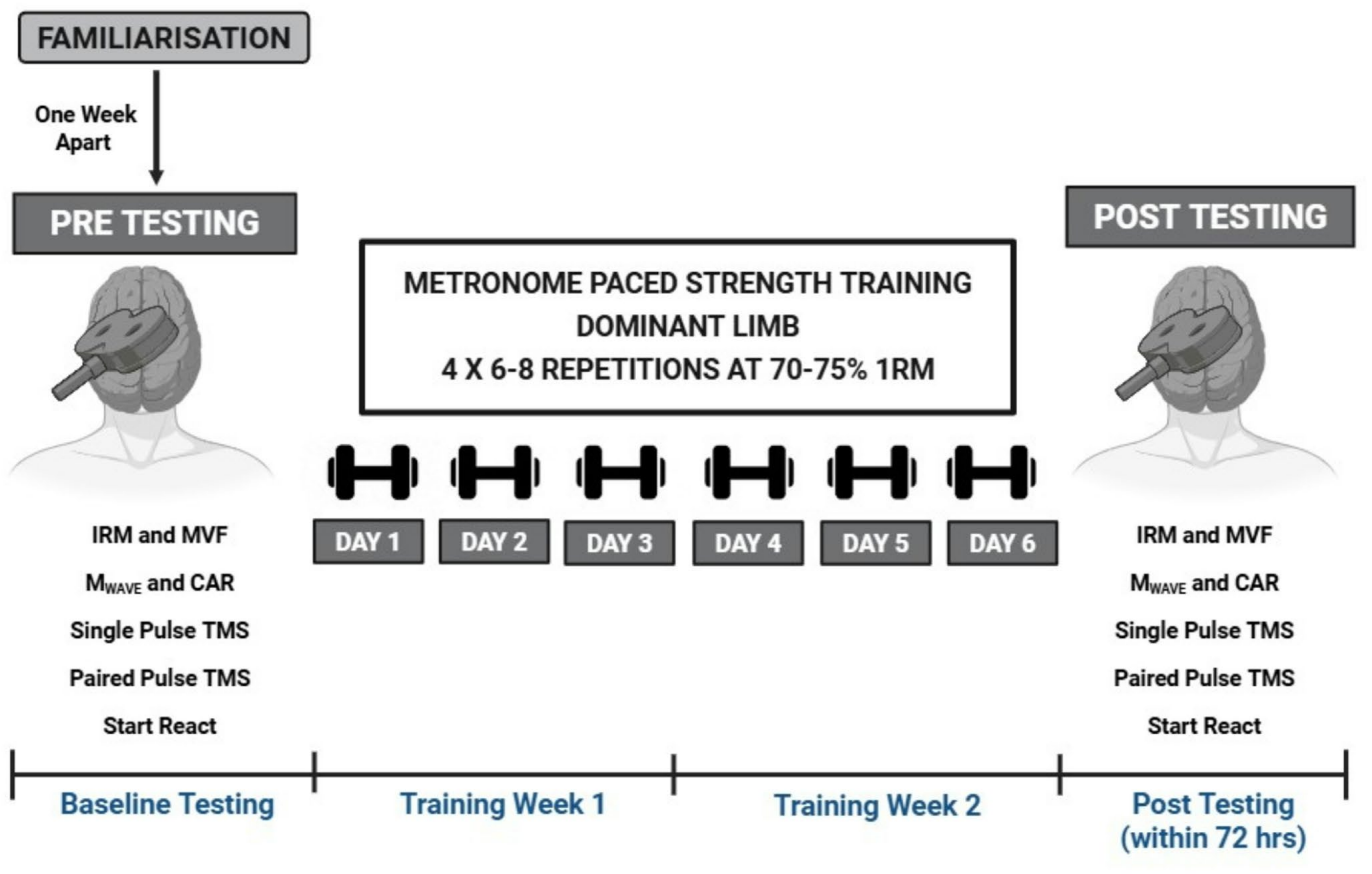
Schematic representation of the experimental design. Participants completed a familiarisation session followed by baseline (pre-training) testing conducted one week later. Participants then performed two weeks of metronome-paced strength training (MPST) of the dominant limb consisting of six training sessions Neurophysiological and strength assessments were performed at baseline and repeated following the intervention, with post-testing conducted within 72 h after the final training session. *1RM* one repetition maximum, *MVF* Maximum Voluntary Force, *MWAVE* Maximum compound potential, *CAR* Central Activation Ratio; *TMS* Transcranial Magnetic Stimulation, *SICI* Short interval intracortical inhibition, *ICF* Intracortical facilitation, *RT* Reaction Time, *SR*_*Effect*_ StartReact Effect, *RFD*: Rate of Force Development

Participants completed six supervised unilateral strength training sessions over two weeks (three sessions per week), commencing one week after familiarisation. Training sessions were separated by a minimum of 48 h. Neurophysiological and behavioural assessments (TMS, strength and StartReact) were obtained at two time points, baseline (Pre) and post (within 72 h of the final training session). All testing sessions were scheduled at the same time of day to minimise circadian variability, and participants were instructed to avoid caffeine and alcohol on testing days, abstain from strenuous physical activity throughout the intervention, and maintain a consistent dietary routine. In summary, the experimental timeline comprised an initial familiarisation session (one week prior to baseline testing), followed by baseline (Pre) testing, six unilateral strength training sessions performed over two weeks, and post-intervention testing conducted within 72 h of the final training session (Fig. 1).

Dynamic strength of the elbow flexors was assessed in the dominant arm using the 1-RM test. Participants first estimated their 1-RM, then performed a standard biceps curl with a dumbbell while standing against a wall to maintain form and minimise compensatory movements. A three-minute rest was provided between sets to reduce fatigue and maximise force output. The load was increased in 0.5 kg increments until failure, with the heaviest successful lift recorded as the 1-RM (Akalu et al. 2024; Siddique et al. 2024). Most participants achieved their 1-RM within three to five attempts. The 1-RM of the trained arm was determined to establish the training load. Relative strength for 1-RM was calculated by normalising force values to body mass at baseline. To avoid acute exercise effects and ensure consistency across sessions, 1-RM testing was conducted at baseline, and prior to training during mid- and post-intervention visits. Root-mean-square EMG (rmsEMG) was simultaneously recorded during all 1-RM assessments .

Following baseline measurements, participants completed six sessions of high-intensity unilateral strength training of their dominant arm over two weeks (three sessions per week). Each session consisted of four sets of 6–8 biceps curls at 70–75% of the individual’s 1-RM. A metronome was used to regulate the tempo of each repetition (3 s concentric, 4 s eccentric), and a two-minute rest was provided between sets to minimise fatigue (Siddique et al. 2024). The training load was progressively increased throughout the intervention once participants were able to complete repetitions with proper technique. Training load-volume was also calculated for each participant using the following formula:

$${\rm{Training}}~{\rm{Load}}~{\rm{Volume}} = \left( {{\rm{Sets~}}\; \times \;{\rm{Repetitions}}\; \times \;{\rm{Load}}} \right)$$

### Surface electromyography (sEMG) and Maximum voluntary force (MVF)

sEMG activity of the biceps brachii in the trained arm was recorded during strength testing at all time points (Pre, Week 1, Week 2, and Post). Bipolar Ag–AgCl electrodes were placed 2 cm apart over the muscle belly, one-third of the distance from the cubital fossa, following SENIAM guidelines (Hermens et al. 2000). The skin was prepared by light abrasion and cleansing with 70% isopropyl alcohol to reduce impedance and improve signal quality (Gilmore and Meyers 1983). A grounding strap was secured around the wrist. sEMG signals were amplified (×1000), band-pass filtered (13–1000 Hz), and digitised at 2 kHz. Data were recorded and analysed using the PowerLab 4/26 system (AD Instruments, Bella Vista, Australia).

MVF of the elbow flexors was assessed in the trained arm. Participants were seated with their elbows flexed at 90° and forearms supinated. A force transducer (Futek Force Transducer LSB302, Melbourne, Australia) was positioned at the wrist level on the forearm. Participants were instructed to perform a maximal isometric contraction of the elbow flexors, maintaining maximal effort for 3 s. Real-time force feedback was displayed on a monitor positioned 1 m in front of the participant. Three trials were performed, with a 3-minute rest between trials to minimise fatigue (Akalu et al. 2024; Siddique et al. 2024). If force output between two trials varied by more than 5%, a third trial was conducted. The highest force value across trials was recorded as the participant’s MVF and used to calculate the 10% target force level for subsequent neurophysiological assessments. MVF testing was conducted at all time points (Pre, Week 1, Week 2, and Post) to track changes in isometric force. Relative MVF, normalised to body mass, was recorded at baseline, and rmsEMG activity during MVF testing was recorded at all testing sessions.

### Motor nerve stimulation and Central activation ratio (CAR)

Peripheral muscle responses in the trained bicep brachii were assessed using supramaximal electrical stimulation of the brachial plexus at Erb’s point (DS7A; Digitimer, UK). Stimulation was delivered via 3.2 cm round self-adhesive electrodes, with the cathode placed over the supraclavicular fossa and the anode on the acromion. Beginning at 20 mA, stimulus intensity was increased in 10 mA increments until a plateau in the evoked sEMG M-wave response was observed. To ensure maximal activation, stimulation intensity was increased by an additional 20% above the plateau level. At this intensity, three single pulses were delivered at 5–8 s intervals. The largest peak-to-peak M-wave amplitude was recorded as the maximal M-wave (M_MAX_). This procedure, which demonstrates high reliability (ICC = 0.92) (Walker et al. 2013), was performed at Pre and Post (after 2 weeks of training) to evaluate changes in peripheral muscle excitability.

Neural drive to the trained biceps brachii was further assessed using the CAR. During a near-maximal isometric contraction (> 90% MVF), a supramaximal stimulus (M_MAX_ intensity, 200 µs pulse width) was delivered to Erb’s point to evoke a superimposed twitch. CAR was calculated as the ratio of voluntary force to the force generated during the superimposed twitch. This assessment was performed at Pre and Post training to evaluate changes in voluntary activation. To quantify overall neural drive to the elbow flexors, CAR (Knight and Kamen 2001) was calculated as follows:

$${\rm{CAR}}~\left( \% \right) = \left( {\frac{{{\rm{Maximal}}~{\rm{Force}}}}{{{\rm{Maximal}}~{\rm{Force}} + {\rm{ITT}}}}} \right)~ \times 100$$

Here, maximal force represents the MVF achieved during maximal isometric testing, while maximal force plus ITT (Interpolated Twitch Technique) represents the additional force generated by the supramaximal stimulus during the contraction.

### Transcranial magnetic stimulation (TMS)

TMS was used to assess cortical and corticospinal excitability at Pre and Post 2 weeks of strength training. Stimulation was delivered via a BiStim unit connected to two Magstim 200^2^ stimulators (Magstim Co., Dyfed, United Kingdom) to elicit motor-evoked potentials (MEPs) from the trained bicep brachii muscle. A 90 mm circular coil was positioned at a 45° angle to induce a posterior-to-anterior current flow in the primary motor cortex (M1). Stimulation sites were localised using the international 10–20 EEG system as a reference for the biceps representation (Herwig et al. 2003). The motor hotspot for the biceps brachii was identified as the coil position that elicited the largest and most consistent MEPs. This location was marked on the scalp and recorded to ensure consistent coil placement across all testing sessions. Following hotspot identification, active motor threshold (AMT) was determined as the lowest stimulation intensity required to evoke MEPs ≥ 200 µV in at least 5 out of 10 consecutive trials during a 10% MVF isometric contraction (Rossini et al. 2015; Weier et al. 2012). AMT was reassessed and adjusted, if necessary, at the Post time point. Single-pulse TMS was delivered at 100%, 130%, 150%, and 170% of AMT, with five stimuli per intensity and a 10-second interstimulus interval, synchronised to a metronome (Kidgell et al. 2010). Consistent pre-stimulus muscle activation was verified by measuring the rmsEMG in the 100 ms window preceding each stimulus. CSE was assessed by measuring MEP amplitudes, while corticospinal inhibition was quantified via the cortical silent period (cSP), which reflects GABA_B_-mediated inhibitory processes. Cortical silent-period onset was defined relative to the timing of the stimulus artefact. Offset was defined as the point at which EMG activity returned to a stable pre-stimulus level. To identify this, horizontal cursors were placed at the maximum and minimum pre-stimulus EMG amplitudes, and silent-period termination was taken as the time at which post-stimulus EMG re-crossed this range and resumed consistent activity (Damron et al. 2008). Participants maintained a 10% MVF isometric contraction during all assessments.

Intracortical inhibitory and facilitatory mechanisms were assessed using paired-pulse TMS protocols. Short-interval intracortical inhibition (SICI), reflecting GABA_A_-mediated inhibition, and intracortical facilitation (ICF), reflecting glutamatergic excitatory activity, were measured using a conditioning stimulus at 80% AMT and a test stimulus at 130% AMT. The interstimulus interval (ISI) was set to 3 ms for SICI and 10 ms for ICF (Mason et al. 2020; Siddique et al. 2024). All TMS data were acquired and analysed using LabChart™ software (version 8.1.24, ADInstruments, Bella Vista, Australia).

TMS data were analysed to assess EMGrms activity in the trained biceps brachii during the 100 ms prior to each stimulus. Trials with pre-stimulus EMGrms exceeding 5 ± 1% of maximal rmsEMG were excluded. MEP peak-to-peak amplitudes at each stimulation intensities (100%-170%AMT) were extracted, normalized to M_MAX_, and multiplied by 100 to account for peripheral muscle changes. The silent period duration was assessed using single-pulse TMS at 130–170% AMT, determined by visual inspection, with onset marked from stimulus delivery and cessation defined by the return of sEMG activity to pre-stimulus levels. All cSP analyses were performed by an investigator blinded to group allocation and time point. SICI and ICF were expressed as a percentage of the unconditioned single-pulse MEP amplitude (Siddique et al. 2024). Specifically, the MEP amplitudes for SICI and ICF were divided by the MEP amplitude elicited through single-pulse TMS at 130% AMT and multiplied by 100.

### StartReact protocol

The StartReact protocol (Tapia et al. 2022) was employed to assess RST activity in the trained arm. This validated approach evaluates the activity of the pontomedullary reticular formation by measuring reaction time (latency) to a pre-planned motor task in response to visual and auditory cues (Baker and Perez 2017; Tapia et al. 2022).

Participants were seated comfortably with relaxed shoulders, elbows flexed at 90°, and forearms in a supinated position. A force transducer was placed at the forearm to measure force output. Participants were instructed to rapidly flex their elbow against the transducer in response to an LED light positioned one meter in front of them at eye level. Prior to data collection, practice trials involving rapid contractions and exposure to auditory stimuli were conducted to ensure familiarity. The protocol included three randomized conditions: (1) LED light alone for visual reaction time (VRT), (2) LED light plus a soft sound (80 dB, 500 Hz, 50 ms) for visual-acoustic reaction time (VART), and (3) LED light plus a loud sound (115–120 dB, 500 Hz, 50 ms) for visual-startle reaction time (VSRT), consistent with established StartReact paradigms showing that auditory stimuli (≥ 110 dB) are typically required to elicit startle-triggered motor responses (Valls-Solé et al. 1995; Carlsen et al. 2004). Auditory stimuli were delivered via a speaker placed one meter behind the participant. Thirty trials were performed with 3–6 s intervals between each to minimize predictability and reduce habituation.

In addition to reaction time, the rate of force development (RFD) was measured at two-time intervals following force onset: 0–50 ms and 50–100 ms, based on previous methodologies (Akalu et al. 2024; Colomer-Poveda et al. 2023). Pilot testing demonstrated strong reliability for the StartReact measures, with intraclass correlation coefficient (ICC) values of 0.72 for VRT, 0.75 for VART, and 0.82 for VSRT. These findings align with Colomer-Poveda et al. (2023), further supporting the validity of the protocol.

StartReact data were processed using a custom macro in LabChart software (ADInstruments, Bella Vista, Australia). Reaction time was defined as the onset latency of sEMG activity in the biceps brachii. Onset was identified when the rectified signal exceeded ± 3 standard deviations above baseline, calculated from a 200 ms pre-stimulus window (Akalu et al. 2024). All trials were manually reviewed to exclude any artefact-related errors in sEMG or force detection. RST excitability was inferred from the degree of reaction time reduction in response to the startle stimulus (Rothwell 2006; Tapia et al. 2022; Valls-Solé et al. 1999). The StartReact Effect (SR_Effect_) was calculated as the difference between VART and VSRT:

$${\rm{S}}{{\rm{R}}_{{\rm{Effect}}}} = \left( {{\rm{VART}} - {\rm{VSRT}}} \right)$$

A larger SR_Effect_ indicates greater excitability of the RST, as the loud acoustic stimulus accelerates the motor response via subcortical pathways, compared to the corticospinal-driven response to softer cues (Carlsen and Maslovat 2019; Germann and Baker 2021; Sangari and Perez 2020). Finally, RFD was calculated as the slope of the force-time curve over the 0–50 ms and 50–100 ms intervals following force onset. Force onset was defined as the point at which the signal exceeded three standard deviations above baseline activity, measured over the same 200 ms pre-stimulus period (Akalu et al. 2024; Colomer-Poveda et al. 2023; Walker et al. 2024).

### Statistical analysis

Statistical analyses were conducted using SPSS software (IBM SPSS Statistics, version 26, New York, USA), and graphical representations were generated using GraphPad Prism (GraphPad Software Inc., San Diego, CA, USA). The normality of the data was assessed using the Shapiro-Wilk test. For data that violated normality assumptions, residual distributions were visually inspected using histograms and assessed for homoscedasticity, in line with established guidelines (Garson 2019). Independent samples t-tests were used to evaluate the differences in physical characteristics and neurophysiological measures between the two groups at baseline.

To assess changes in strength (1RM, MVF), cortical (SICI, ICF), corticospinal (CSE, cSP), central activation ratio (CAR), and reticulospinal (reaction time, SR_Effect_ and RFD) measures across groups and time points, a Linear Mixed Model with Repeated Measures (LMM_RM_) was used, employing Maximum Likelihood (ML) estimation (Wilkinson et al. 2023). This model was chosen to account for repeated measurements, missing data, and inter-individual variability. It included both fixed and random effects, with a random intercept specified at the participant level to account for within-subject variability and repeated observations. Trial-level data were entered into the model to preserve granularity and enhance statistical power. Fixed effects included time (Pre, Week 1, Week 2, Post), age group (Older and Young adults), stimulation intensity or maximal stimulator output (MSO) (100%–170% AMT), StartReact cue condition (VRT, VART, VSRT), and time window (0–50 ms and 50–100 ms for RFD), along with their relevant interactions (e.g., AgeGroup × Time, AgeGroup × Time × MSO, AgeGroup × Time × StartReact cue, AgeGroup × Time × StartReact cue × Time Window).

Post hoc comparisons were adjusted using Bonferroni correction for multiple comparisons. Assumptions of linearity and normality were checked via visual inspection of scatterplots and residual distributions. Effect sizes were calculated for within- and between-group comparisons over time using Hedges’ g, with the following cut-offs for interpretation: small effects < 0.2, moderate effects = 0.2–0.8, and large effects > 0.8 (Hedges and Olkin 2014). Statistical significance was set at *p* < 0.05 for all analyses. Model-derived outcomes are reported as estimated marginal means (EMMs) with 95% CI, unless otherwise specified. Raw means ± SD are also reported where applicable. Figures display raw individual values with 95% CI, whereas tables report fixed-effect estimates from the mixed-model analyses (Fig. 1).

##  Results

### Baseline electrophysiological measures

Both older and young adults completed the study with no withdrawals or reported adverse events. No between group differences were found for 1-RM strength (*p* = 0.882, g = 0.06), 1-RM relative strength (1-RM/Body weight) (*p* = 0.503, g = 0.31), MVF (*p* = 0.342, g = 0.38), MVF Relative Strength (MVF/Body weight) (*p* = 0.085, g = 0.70), MVFrms EMG (%M_MAX_) (*p* = 0.188, g = 0.54), AMT (*p* = 0.232, g = 0.48), cSP(*p* = 0.815, g = 0.09), ICF (*p* = 0.756, g = 0.01), CAR (*p* = 0.072, g = 0.88), M_MAX_ amplitude (*p* = 0.286, g = 0.43), Training Volume (*p* = 0.941, g = 0.03), or SR_Effect_ (*p* = 0.111, g = 0,64). However, significant baseline differences were identified for 1-RM rmsEMG (%M_MAX_) (*p* = 0.016, g = 0.83) and SICI (*p* = 0.025, g = 0.95), with young adults exhibiting greater 1-RM rmsEMG and reduced intracortical inhibition (i.e., greater SICI) compared to older adults (Supplementary Table 1).

### Training induced strength adaptations

Analysis of 1-RM strength revealed a significant main effect for **Time** (F _(1,25)_ = 98.5, *p* < 0.001). There were no main effects of **AgeGroup** (F _(1,25)_ = 0.040, *p* = 0.842) or **Time** × **AgeGroup** interaction (F _(1, 25)_ = 0.210, *p* = 0.651). Both older and young adults demonstrated significant increases in 1-RM following the training period compared to baseline (all *p* < 0.001).

**Fig. 2:**
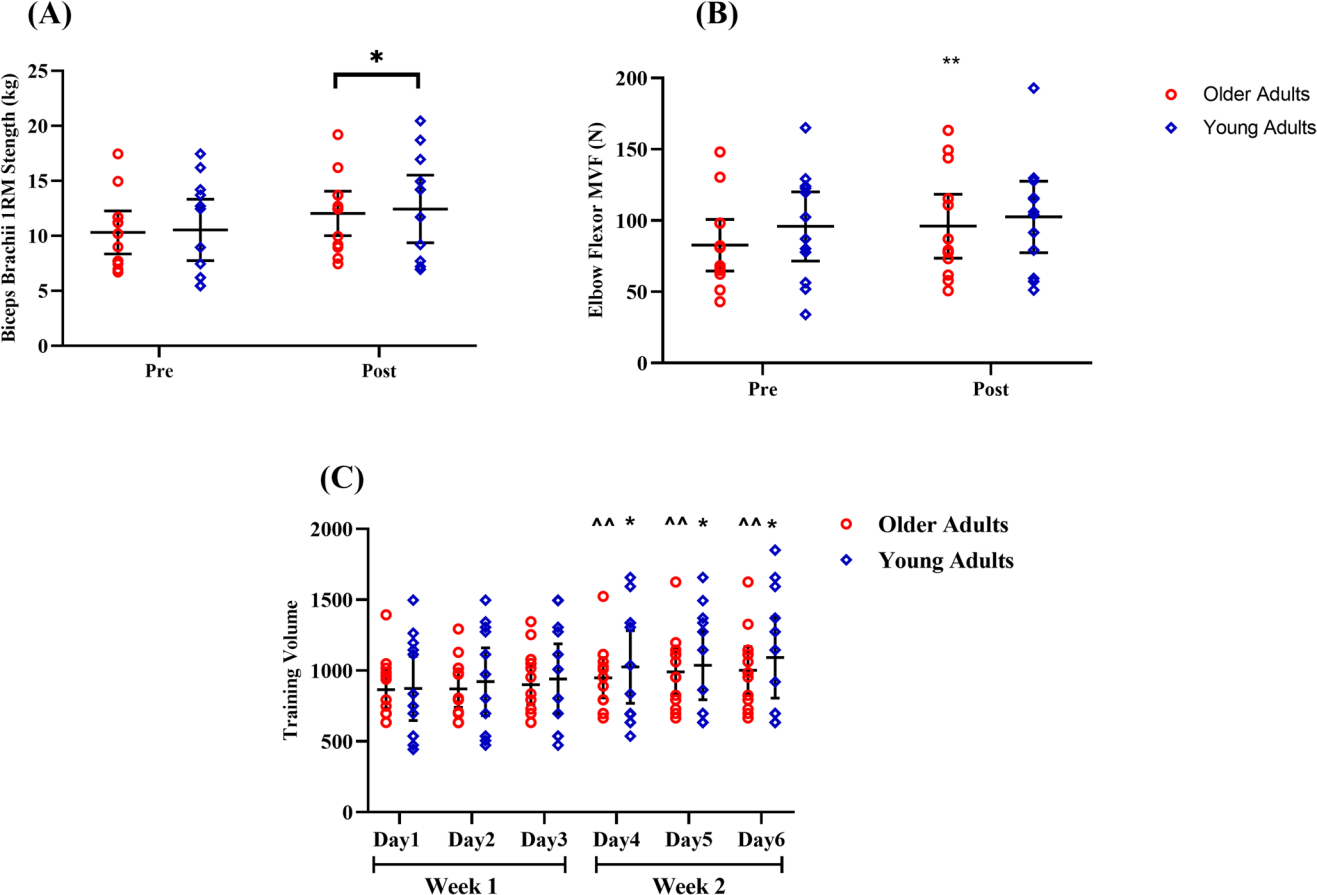
**A** One-repetition maximum (1RM) strength, **B** maximal voluntary force (MVF), and **C** training volume (mean ± 95% CI) of the trained biceps brachii in older and young adults. *p < 0.001 indicates a significant main effect of time from pre in both groups. ** *p* < 0.001 indicates a significant main effect of time from pre-post for MVF in older adults, ^^*p* < 0.01 indicates a significant increase in training volume from pre in older adults. *p < 0.05 indicates a significant increase in training volume from pre in young adults

Similarly, for MVF, a significant main effect of **Time** was observed (F _(1,25)_ = 12.65, *p* < 0.001), with no significant **AgeGroup** effect (F _(1,25)_ = 0.476, *p* = 0.497) or **Time × AgeGroup** interaction (F _(1,25)_ = 0.149, *p* = 0.245). Post hoc comparisons showed significant increases in MVF post training relative to baseline for the older adults only (*p* < 0.001; Fig. 2, Supplementary Table 1).

### Training effects on corticospinal excitability (MEP) and inhibition (cSP)

**Fig. 3:**
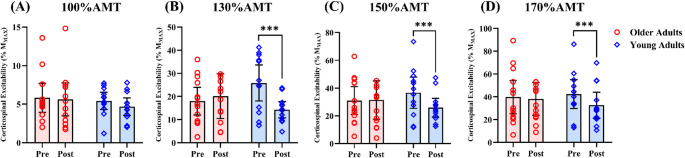
Motor-evoked potential (MEP) amplitude of the trained biceps brachii measured at **A** 100% AMT, **B** 130% AMT, **C** 150% AMT, and **D** 170% AMT, expressed as a percentage of M_MAX_ (Mean ± 95% CI) in older and young adults. ***p < 0.001 indicates a significant decrease from pre-in young adults

For MEP amplitude, significant main effects were observed for **MSO** (F _(3,975)_ = 300.05, *p* < 0.001), **Time** (F _(1,975)_ = 24.43, *p* < 0.001) and **Time × AgeGroup** interactions (F _(1,975)_ = 28.22, *p* < 0.001). The main effect of **AgeGroup** was not significant (F _(1,25)_ = 0.046, *p* = 0.831). Post hoc comparisons for time revealed no significant changes in MEP amplitude from Pre to Post in older adults across all MSOs. In contrast, post hoc comparisons for **Time × AgeGroup** revealed that young adults exhibited significant reductions in MEP amplitude at 130–170% AMT following training (MD = 9.8 to 11.54, all *p* < 0.001, g = 0.50 to 1.18) (Fig. 3).

**Fig. 4:**
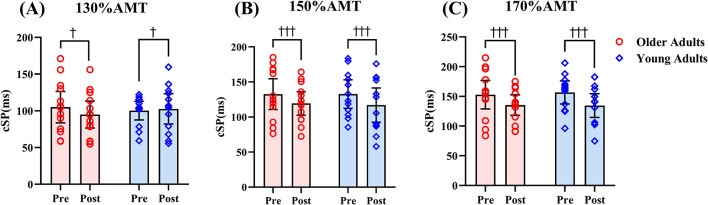
Cortical silent period (CSP) duration (ms) of the trained biceps brachii measured at **A** 130% AMT, **B** 150% AMT, and **C** 170% AMT, expressed as mean ± 95% CI in older and young adults. ^†^*p* < 0.05 and ^†††^*p* < 0.001 indicate a significant main effect of time (collapsed across groups) for cSP at 130, 150, and 170% AMT

Furthermore, for cSP, significant main effects were found for **MSO** (F _(2,713) =_ 221.992, *p* < 0.001), **Time** (F _(1,713)_ = 54.197, *p* < 0.001) and **MSO × Time interactions** (F _(2,713) =_ 3.829, *p* = 0.022) indicating an overall reduction in cSP duration from Pre to Post across participants. However, there was no significant main effect of **AgeGroup** (F_1,25_ = 0.000, *p* = 0.991) or **Time × AgeGroup** interaction (F _(1,713)_ = 3.108, *p* = 0.078) suggesting that the decrease in cSP was comparable between younger and older adults (Fig. 4, Supplementary Table 2).

### Training effects on intracortical changes (SICI and ICF)

**Fig. 5:**
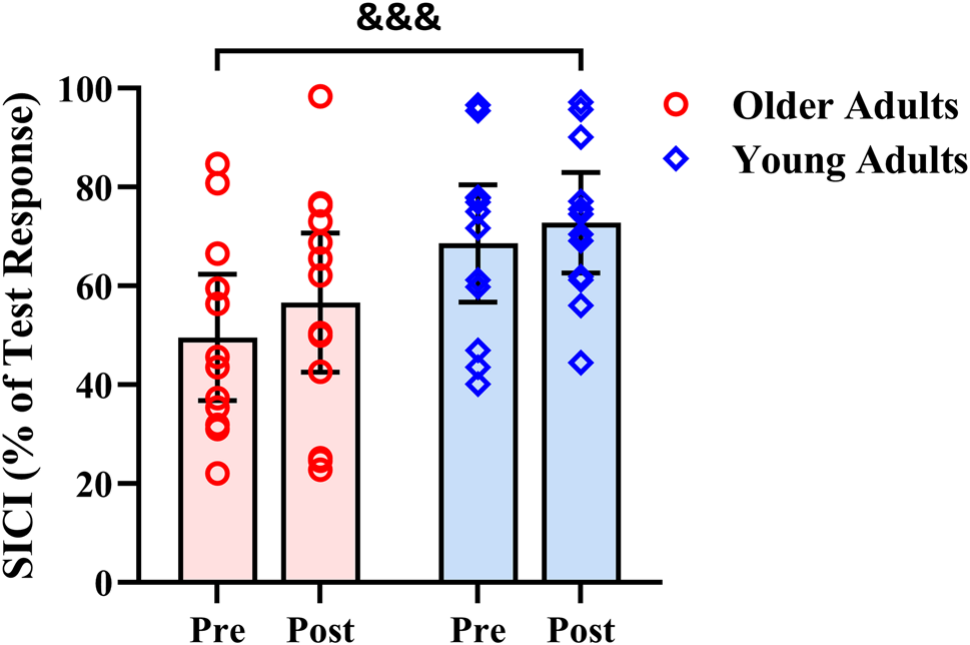
Short-interval intracortical inhibition (SICI; mean ± 95% CI) of the trained biceps brachii in older and young adults. ^&&&^*p* < 0.001 indicates a significant main effect of group between older and young adults. This main effect indicates reduced intracortical inhibition in younger compared with older adults, independent of training

There was no significant main effect of **Time** (F _(1, 201.22)_ = 3.59, *p* = 0.059), although SICI showed a small increase post (Estimate = 6.43, 95% CI [2.00, 14.86]). There was, however, a significant main effect of **AgeGroup** (F _(1, 22.70)_ = 16.36, p = < 0.001), with older adults demonstrating lower SICI values (Estimate = − 19.40, 95% CI [− 30.69, − 8.12], g = 0.31) compared with younger adults (Fig. 5). The **Time × AgeGroup** interaction was not significant (F _(1, 201.22)_ = 0.06, *p* = 0.811), demonstrating that changes over time were similar across groups. For ICF there was no significant main effect of **Time** (F _(1, 144.90)_ = 0.269, *p* = 0.605), **AgeGroup** (F _(1, 25.90)_ = 0.14, *p* = 0.710) or **Time × AgeGroup** (F _(1, 144.90)_ = 0.65, *p* = 0.423).

### Training effects on peripheral excitability (M_MAX_) and central activation ratio (CAR)

**Fig. 6:**
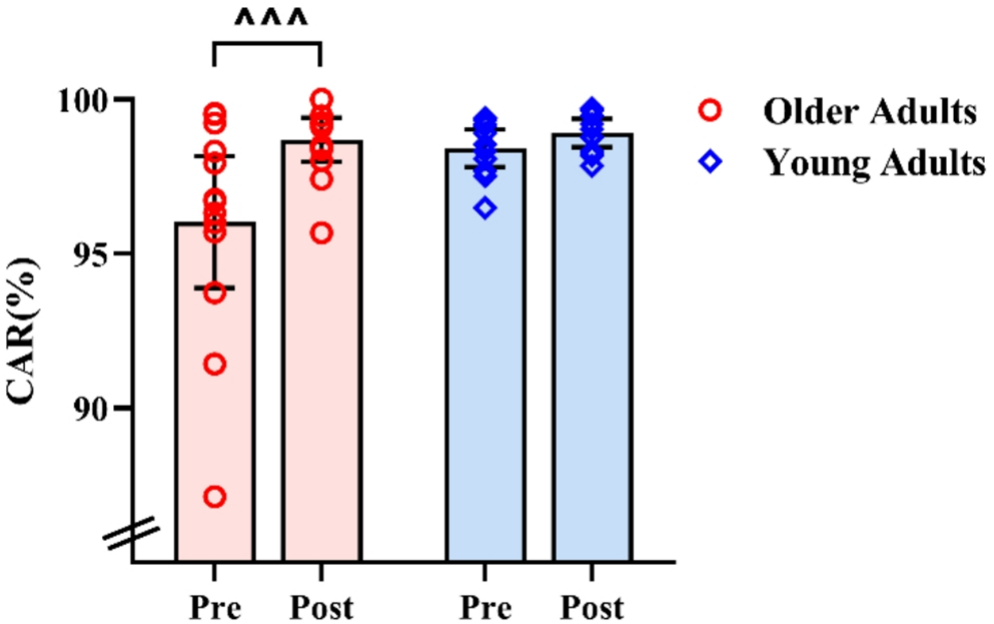
Central activation ratio (CAR; mean ± 95% CI) of the trained biceps brachii in older and young adults. ^^^*p* < 0.001 indicates a significant increase in CAR from pre- to post-training in older adults, reflecting enhanced voluntary neural drive following training, with no corresponding change observed in young adults

There was no significant main effect of **Time** (F _(1, 25)_ = 3.90, *p* = 0.060), although M_MAX_ showed a small decrease from pre to post (Estimate = − 1.55, 95% CI [− 3.27, 0.16]). The main effect of **AgeGroup** was also not significant (F _(1, 25)_ = 2.88, *p* = 0.102), with older adults demonstrating slightly lower M_MAX_ values than younger adults, but the difference did not reach statistical significance (Estimate = − 2.68, 95% CI [− 5.64, 0.28]). The **Time × AgeGroup** interaction was not significant (F _(1, 25)_ = 0.51, *p* = 0.483), indicating that neither age group displayed a change in M_MAX_ across the testing period (Fig. 6).

A linear mixed model assessed the effects of **Time** (PRE, POST), **Group** (Old, Young), and their interaction on CAR. There was a significant main effect of **Time** (F _(1, 24)_ = 11.78, *p* = 0.002), indicating a reliable change in neural drive across the testing period. The main effect of **AgeGroup** approached significance (F _(1, 24)_ = 4.10, *p* = 0.054), with young adults demonstrating slightly higher CAR values overall. However, the **Time × AgeGroup** interaction was significant (F _(1, 24)_ = 5.55, *p* = 0.027), indicating that the pattern of change across time differed between groups. Parameter estimates showed that older adults exhibited a greater increase in CAR over time relative to young adults (SupplementaryTable 2).

### Training effects on subcortical and behavioural measures (Start React Test)

**Fig. 7:**
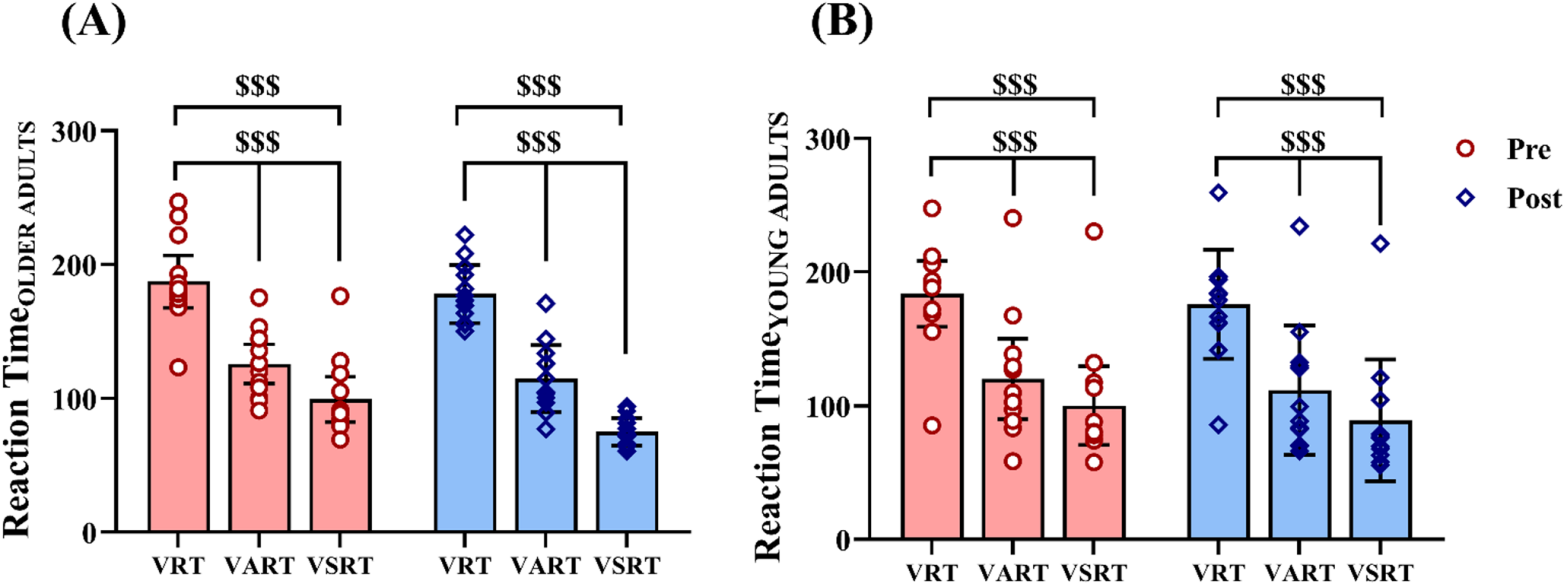
Reaction times (mean ± 95% CI) of the trained biceps brachii during visual reaction time (VRT; visual-only), visual–auditory reaction time (VART; light + soft sound), and visual–startle reaction time (VSRT; light + startling sound) in **A** older and **B** young adults across the two-week training intervention. Significant differences between StartReact cues are indicated by ^$$$^*p* < 0.001

There was a significant main effect of **Condition** (F _(2, 125.01)_ = 272.01, *p* < 0.001), indicating differences in reaction time across conditions. Post hoc analysis showed that VRT elicited the slowest responses (M = 181.45 ms), VART produced intermediate latencies (M = 118.10 ms), and VSRT yielded the fastest responses (M = 90.71 ms). All pairwise differences were statistically significant (all, *p* > 0.05). A significant main effect of **Time** emerged (F _(1, 125.77)_ = 12.26, *p* = 0.001), reflecting faster responses at Post (M = 124.32 ms) than Pre (M = 135.86 ms). This improvement was consistent across conditions. In contrast, **AgeGroup** did not influence reaction time (F _(1, 24.99)_ = 0.00, *p* = 0.993, with similar mean estimates for Old (M = 130.04 ms) and Young adults (M = 130.14 ms). No interaction terms reached significance (all *p* > 0.24), indicating that task difficulty effects and practice-related improvements were consistent across both age groups. Condition-specific means across **Time × AgeGroup** showed the same pattern: VRT remained the slowest and VSRT the fastest at both time points, with modest pre-to-post reductions in latency. However, none of these descriptive differences were supported by significant interactions. Overall, the data demonstrate strong condition effects, moderate practice effects, and no evidence of age-related differences in reaction time performance (Fig. 7).

**Fig. 8:**
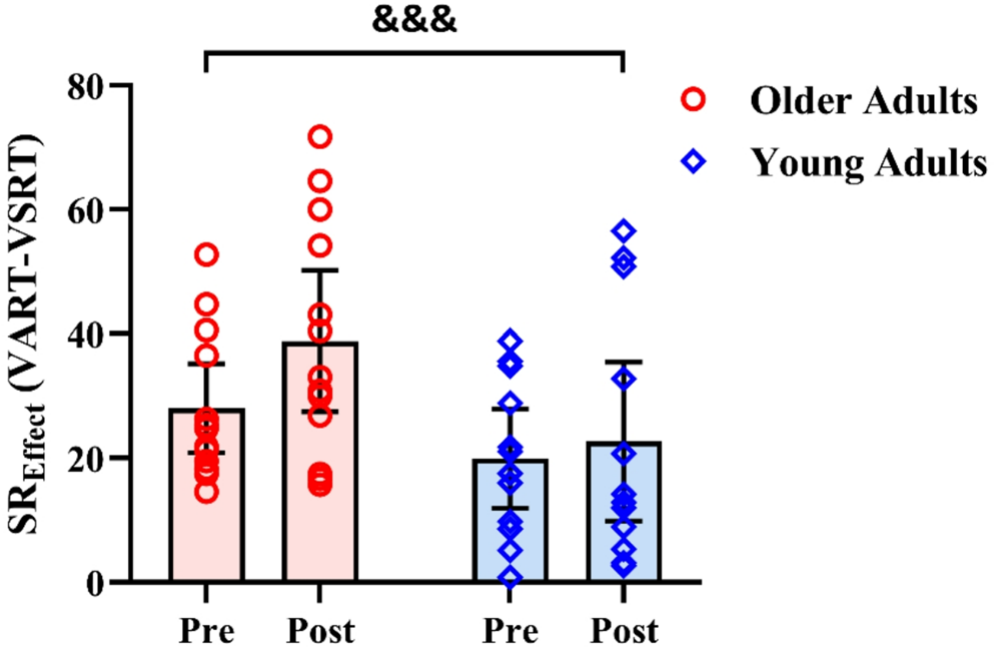
StartReact effect (mean ± 95% CI) in the trained biceps brachii of older and young adults. ^&&&^*p* < 0.001 indicates a significant main effect of group, with older adults exhibiting a consistently larger StartReact effect than young adults across time points, indicating a greater reduction in reaction time under startle conditions

A separate linear mixed model analysed the effects of **Time** (Pre, Post), **AgeGroup** (Old, Young), and their interaction on **SR**_**Effect**_. The main effect of **Time** was not significant (F _(1, 25)_ = 2.77, *p* = 0.109). There was a significant main effect of **AgeGroup** (F _(1, 25)_ = 6.59, *p* = 0.017), with Older adults showing larger **SR**_**Effect**_ (M = 33.45, SE = 3.27) than Young adults (M = 21.33, SE = 3.41). Pairwise comparisons confirmed a mean difference of 12ms in the **SR**_**Effect**_ (95% CI [2.40, 21.85], *p* = 0.017), indicating a markedly greater effect in older adults, consistent with increased reticulospinal excitability relative to young adults. The **Time × AgeGroup** interaction was not significant (F _(1, 25)_ = 0.97, *p* = 0.334), indicating that pre-to-post changes did not differ reliably between age groups. Descriptively, the Older group showed a larger increase (Pre: 28.05 vs. Post: 38.86) relative to the Young group (Pre: 19.95 vs. Post: 22.72), but within-group pairwise tests did not reach statistical significance (Old: *p* = 0.067; Young: *p* = 0.642) (Fig. 8; Supplementary Table 3).

**Fig. 9:**
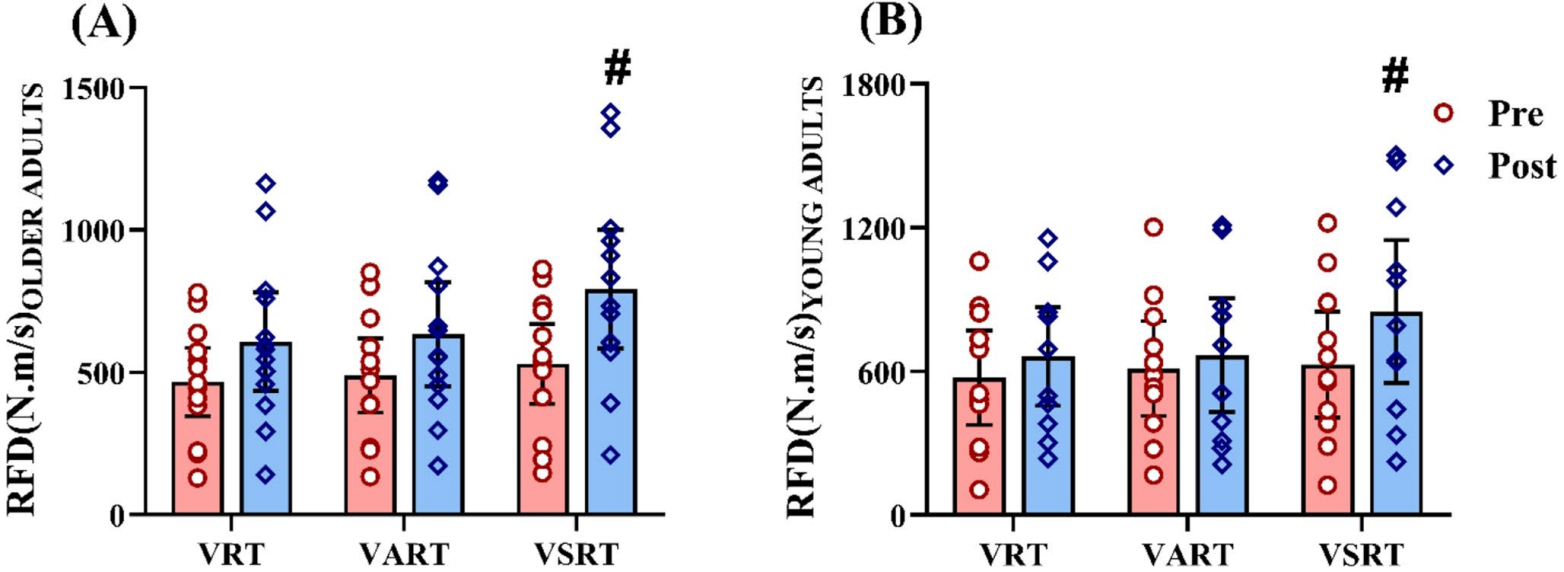
Force production (mean ± 95% CI) of StartReact responses in the trained biceps brachii of older and young adults. The rate of force development (RFD) is shown for the 0–50 ms time window. Responses were recorded under three cue conditions: visual-only (VRT), light + soft sound (VART), and light + startling sound (VSRT). #*p* < 0.001 denotes significant increase in 0–5.ms RDF from pre-post in older and young aduluts

A Linear mixed-model analysis evaluated the effects of **Time Window** (TW: 0–50 ms, 50–100 ms), **Condition** (VRT, VART, VSRT), **Time** (Pre, Post), and **AgeGroup** (Old, Young) on RFD. There was a strong main effect of **TW** (F _(1, 264)_ = 513.91, *p* < 0.001), with substantially greater RFD in the 0–50 ms window (M = 625.87) relative to 50–100 ms (M = 252.29), reflecting the rapid early rise in force. Further, a significant main effect of **Condition** was observed (F _(2, 264)_ = 4.40, *p* = 0.013), with the lowest RFD during VRT (M = 416.73), moderate values in VART (M = 427.41), and the highest values in VSRT (M = 473.10), indicating enhanced early-phase force production under StartReact like conditions. There was also a significant main effect of **Time** (F _(1, 264)_ = 24.09, *p* < 0.001), with higher RFD at Post (M = 479.52) relative to Pre (M = 398.64), showing improved early-phase force generation following strength training. In contrast, **AgeGroup** was not significant F _(1, 24)_ = 0.37, *p* = 0.549), and estimated means showed only modest differences between Old (M = 415.92) and Young (M = 462.24) adults. Several interactions reached significance, including **TW × Condition** (F _(2, 264)_ = 5.96, *p* = 0.003, and **TW × Time** (F _(1, 264)_ = 18.74, *p* < 0.001), indicating that task demands and training effects differentially influenced early time windows. No interactions involving **AgeGroup** were significant. Collectively, the RFD results demonstrate significant early-phase force production differences across conditions, training-related increases in RFD, and no evidence of age-related deficits in RFD (Fig. 9).

## Discussion

This study provides the first concurrent evaluation of corticospinal and reticulospinal adaptations following short-term, high intensity strength training in older and young adults. Both groups exhibited significant improvements in strength, with 1-RM increasing after two weeks, confirming that short-term training elicits early functional gains irrespective of age. Corticospinal responses diverged, with young adults showing reduced MEP amplitudes post-training, while older adults showed no measurable change. Despite this, both groups demonstrated shortened cSP duration, reflecting reduced intracortical inhibition within GABAergic circuits. Intracortical measures further confirmed persistent age-related differences, with younger adults showing greater SICI overall, although neither SICI nor ICF was altered by training. Neural drive, indexed by the CAR, increased in older adults only, suggesting an age-dependent enhancement of voluntary activation to support strength gains. Reticulospinal involvement was evident across groups, as shown by faster StartReact responses and pronounced increases in early-phase RFD, both consistent with enhanced subcortical contributions to rapid force production. Collectively, these findings demonstrate that strength training induces early, multi-level plasticity across distinct motor pathways, underscoring the potential of pathway-specific strategies to improve neuromuscular function in ageing populations.

###  Training induced strength gains across age groups

Strength training produced significant increases in both MVF and 1-RM strength in older and young adults, with no significant AgeGroup × Time interaction, indicating comparable relative gains across age groups. These findings in the upper limb align with lower-limb studies reporting early strength improvements irrespective of age (Christie and Kamen 2014; Gomez-Guerrero et al. 2024; Häkkinen et al. 2000; Walker and Häkkinen 2014). Following training, older adults showed an 18% increase in 1-RM strength, while young adults improved by 20%. MVF increased by 16% in older adults, and by 10% in young adults post training. Although these numerical differences were not statistically significant, they are consistent with prior observations that individuals with lower baseline strength exhibit greater relative gains (Ahtiainen et al. 2003; Christie and Kamen 2014) and suggest that older adults remain as responsive as young adults to short-term strength training (Kamen and Knight 2004; Patten et al. 2001). Furthermore, the magnitude of improvement observed within this short intervention mirrors that reported for longer-duration training protocols of up to 10 weeks (Walker and Häkkinen 2014), supporting the view that early-phase strength gains are likely underpinned by neural mechanisms rather than muscular hypertrophy.

### Age related cortical plasticity to strength training

Our findings demonstrate clear age-dependent differences in CSE following short-term strength training. Older adults showed no change in CSE, consistent with evidence that corticospinal responsiveness is reduced with age (Cirillo et al. 2010; Oliviero et al. 2006; Pitcher et al. 2003; Rogasch et al. 2009; Sale and Semmler 2005; Todd et al. 2010). Although baseline MEP amplitudes did not differ between groups, the absence of training-induced modulation in older adults aligns with reports that strength gains can occur without measurable increases in CSE (Christie and Kamen 2014). Reductions in CSE have been observed after longer training periods (Gomez-Guerrero et al. 2024), suggesting that the shorter intervention used here may be insufficient to drive detectable corticospinal adaptations in ageing. Taken together, these observations support the view that the CST shows limited short-term plasticity with ageing, and that early strength gains in older adults may rely on alternative neural pathways.

In young adults, the reduction in MEP amplitude post-training contrasts with meta-analytic evidence of CSE facilitation after short-term strength training (Gómez-Feria et al. 2023; Siddique et al. 2020). However, variability in early corticospinal responses is well documented, with several studies reporting no facilitation or even suppression (Carroll et al. 2002; Coombs et al. 2016b; Jensen et al. 2005; Lee et al. 2009). The decrease in CSE observed here may reflect homeostatic regulation, whereby repeated high-intensity activation reduces excitability to maintain network stability (Müller-Dahlhaus and Ziemann 2015). Importantly, the absence of an increase in CSE should not be interpreted as evidence of reduced neural contribution to strength adaptation, as MEP amplitude reflects only a partial index of corticospinal output and does not directly quantify the total neural drive or its functional contribution to force production following training (Carroll et al. 2002). Spinal processes such as altered motoneurone recruitment or intrinsic excitability, may also contribute, although we did not assess spinal excitability directly.

Modulation of intracortical inhibitory circuits was also evident. Both age groups showed marked reductions in cortical silent period (cSP) duration, a measure largely mediated by GABA_B_ mechanisms (McDonnell et al. 2007; Werhahn et al. 1999). Persistent cSP shortening is well established in young adults following strength training (Coombs et al. 2016a; Hendy and Kidgell 2013; Kidgell et al. 2010; Latella et al. 2012; Mason et al. 2017), and our findings extend this pattern to older adults, indicating that reduced GABA_B_-mediated inhibition is a conserved early adaptation to strength training across age. The cSP and MEP reflect distinct processes (Hallett 1995), so reductions in cortical inhibition can occur independently of changes in CSE. Functionally, cSP shortening may enhance voluntary drive and stabilise motor output during high-force contractions (Stinear et al. 2009). The uniform reduction across age groups suggests that strength training reliably engages inhibitory circuits within the M1, consistent with earlier work showing reduced GABA_B_-mediated inhibition after strength training (Christie and Kamen 2014; Mason et al. 2020). These results highlight preserved GABA-mediated plasticity in older adults and identify it as a potential target for intervention.

By contrast, short-interval intracortical inhibition (SICI), mediated by GABA_A_ receptors (Chu et al. 2008; Kujirai et al. 1993), showed no training-related modulation. Despite this, young adults displayed higher SICI ratios (i.e., reduced inhibition) than older adults, consistent with previous evidence of increased intracortical inhibition with ageing (Opie and Semmler 2014; Peinemann et al. 2001). Thus, while strength training may not influence GABA_A_-mediated inhibition over short time periods, age-related differences in SICI remain evident.

Intracortical facilitation (ICF) was likewise unchanged in both groups. This suggests that glutamatergic excitatory circuits probed by paired-pulse TMS are unlikely to contribute substantially to early strength gains induced by strength training. The literature remains mixed regarding how these circuits adapt (Gómez-Feria et al. 2023; Kidgell et al. 2017; Siddique et al. 2020), and notably, no study has examined ICF responses to strength training in older adults (Siddique et al. 2022). Given potential age-related declines in glutamatergic transmission and their relevance for motor learning and recovery (McEntee and Crook 1993), further work is needed to clarify whether excitatory intracortical pathways support strength adaptation across the lifespan.

###  Subcortical plasticity and functional adaptations to strength training

The reticulospinal tract (RST) provides a major descending pathway for generating force, projecting to spinal motoneurones via mono- and polysynaptic connections (Baker 2011; Baker and Perez 2017; Davidson et al. 2007; Peterson et al. 1975). In contrast to the CST, which has dominated the strength-training literature, the RST remains poorly characterised in humans. To our knowledge, this is the first study to examine reticulospinal responsiveness to short-term strength training in older adults.

Strength training produced a clear reduction in reaction time across participants, with the fastest responses consistently occurring in the VSRT condition, as expected (Davis et al. 1982; Valls-Solé et al. 1995). Notably, older adults showed a markedly greater reduction in startle-evoked responses than young adults (∆24% [g = 1.11] vs. ∆11% [g = 0.23]). The StartReact effect was also larger in older adults (∆39% [g = 0.66] vs. ∆14% [g = 0.16]), indicating a stronger reticulospinal contribution to the release of prepared movements. The shortened startle-evoked reaction times following strength training are consistent with enhanced responsiveness of the reticulospinal tract (Akalu et al. 2023, 2025; Atkinson et al. 2022; Mooney et al. 2023; Tapia et al. 2022). The RST arises from the pontomedullary reticular formation (PMRF), which can trigger rapid, involuntary movement by releasing pre-programmed motor commands when exposed to strong sensory input (Carlsen et al. 2004; Valls-Solé et al. 1999). Strength training may potentiate this pathway by increasing excitability within PMRF neurones and strengthening reticulospinal synapses onto spinal interneurones and motoneurones, thereby lowering the threshold for startle-triggered responses (Davis et al. 1982; Hammond 1973; Nonnekes et al. 2014). These adaptations indicate that short-term strength training can elicit plasticity within subcortical circuits, offering an additional route for enhancing motor output across age. Evidence from non-human primates shows increased reticulospinal excitability after strength training (Glover and Baker 2020), and human studies report greater reticulospinal drive in stronger individuals and following a single session of strength exercise (Akalu et al. 2024, 2025; Maitland and Baker 2021). Together, these findings support the view that strength training can recruit the RST to augment force production.

Improvements in RFD parallel the reaction-time findings and further implicate reticulospinal tract involvement. RFD is considered a sensitive marker of RST activity because it reflects the rapid activation of large, high-threshold motor units (Škarabot et al. 2021, 2022). The increase in RFD observed after strength training likely reflects enhanced recruitment and discharge of these units, which underpin explosive force generation (Aagaard et al. 2002; Del Vecchio et al. 2019; Škarabot et al. 2022; Van Cutsem et al. 1998). Spinal motoneurones act as the final common pathway for force production, integrating descending inputs from both cortical and subcortical sources (Evarts 1968; Riddle et al. 2009). In line with this, startling stimuli amplify early-phase force output by increasing reticulospinal drive to motoneurones (Akalu et al. 2023; Brown et al. 1991; Carlsen et al. 2004; Škarabot et al. 2022; Tapia et al. 2022). Both age groups improved in RFD after strength training across all conditions, with the strongest effects in the 0–50 ms window, reflecting enhanced capacity for rapid force production. Gains were largest when responses were elicited by startling stimuli (Walker et al. 2024). Although older adults displayed lower baseline RFD (529.59 N/s vs. 628.05 N/s in young adults), they showed disproportionately larger improvements following strength training (∆50% [g = 0.86] vs. ∆35% [g = 0.55]). These findings point to the RST as a key locus for early neural adaptation, particularly in older adults. However, the observed improvements may also reflect contributions from spinal-level adaptations and changes in motor unit recruitment or discharge behaviour, which were not directly assessed in the present study. Overall, the results indicate that the RST remains responsive to strength training across the lifespan and may serve as a flexible and robust site of subcortical plasticity.

###  Central and peripheral adaptations to strength training

Clear age-related differences were evident in the adaptation of efferent drive. In older adults, strength training produced substantial increases in the central activation ratio (CAR), indicating improved voluntary activation and greater recruitment of motor units. This is consistent with previous work showing that older individuals often exhibit incomplete activation at baseline, providing greater scope for supraspinal adaptation (Kent-Braun and Ng 1999; Klass et al. 2007; Unhjem et al. 2015). A recent meta-analysis supports this pattern, reporting reliable gains in voluntary drive with strength training in ageing cohorts (Arnold and Bautmans 2014). By contrast, young adults showed no measurable change in CAR, consistent with voluntary activation approaching maximal levels before training, a ceiling effect commonly observed in this population (Carroll et al. 2002; Kamen and Knight 2004). Nonetheless, increases in efferent drive have been demonstrated in young adults when training loads are higher or interventions are longer, indicating that plasticity is preserved but requires a more demanding stimulus (Knight and Kamen 2001; Walker and Häkkinen 2014).

Peripheral excitability, assessed via M_MAX_ amplitude, remained unchanged in both age groups. This suggests that short-term strength training does not substantially modify sarcolemmal or neuromuscular junction excitability within the timeframe studied. These findings align with previous reports showing stable M_MAX_ values following brief training interventions (Lee et al. 2009), reinforcing the idea that peripheral adaptations contribute minimally to early strength gains. Although subtle changes in ion channel function or neuromuscular transmission cannot be entirely excluded (Palmieri et al. 2004), the absence of measurable M_MAX_ modulation supports the conclusion that early improvements in strength are driven primarily by neural adaptations (Aagaard et al. 2002; Carroll et al. 2002).

### Study limitations

While this is the first study to concurrently assess corticospinal and reticulospinal adaptations to short-term MPST in both young and older adults, several limitations warrant consideration. The two-week intervention was sufficient to elicit early-phase neural changes, yet the timeframe may have been insufficient to capture longer-term structural plasticity. Although participants were classified as sedentary, self-reported physical activity may have underestimated incidental movement, potentially influencing baseline motor performance. Despite employing a broad neurophysiological battery, the absence of techniques such as ipsilateral MEPs or cervico-medullary-evoked potentials limited the resolution with which adaptations could be localised within specific descending pathways. Comparisons with other training modalities (e.g., high-load or power-focused protocols) were not included, restricting conclusions regarding the specificity and generalisability of the observed effects. Additionally, while the sample size was modest, this is consistent with the scale of many neurophysiological training studies. The repeated-measures design, combined with linear mixed-effects modelling, strengthens the robustness of the analysis by capturing within-subject changes over time while accounting for inter-individual variability.

## Conclusion

This study demonstrates that short-term strength training elicits distinct, pathway-specific neural adaptations in young and older adults. Although strength gains were comparable, the underlying mechanisms differed: ***young adults*** showed reduced CSE, whereas ***older adults displayed*** greater enhancements in reticulospinal responsiveness and voluntary activation. Across both groups, a consistent shortening of the cortical silent period indicated reduced GABA_B_-mediated inhibition, and increased reticulospinal output provided a common subcortical route for improving force production. These findings highlight the capacity of both cortical inhibitory circuits and subcortical descending pathways to adapt over short timescales and identify the reticulospinal tract as a particularly important contributor to early neural adaptation in ageing. Strength training therefore represents an efficient means of engaging distributed neural mechanisms to enhance neuromuscular function across the lifespan.

## Supplementary Information

Below is the link to the electronic supplementary material.

Supplementary Material 1

## Data Availability

Data for the experiments reported here can be made available upon reasonable request.

## References

[CR1] Aagaard P, Simonsen EB, Andersen JL, Magnusson P, Dyhre-Poulsen P (2002) Neural adaptation to resistance training: changes in evoked V-wave and H-reflex responses. J Appl Physiol 92(6):2309–2318 12015341 10.1152/japplphysiol.01185.2001

[CR2] Ahtiainen JP, Pakarinen A, Alen M, Kraemer WJ, Häkkinen K (2003) Muscle hypertrophy, hormonal adaptations and strength development during strength training in strength-trained and untrained men. Eur J Appl Physiol 89:555–563 12734759 10.1007/s00421-003-0833-3

[CR3] Akalu Y, Frazer AK, Howatson G, Pearce AJ, Siddique U, Rostami M, Tallent J, Kidgell DJ (2023) Identifying the role of the reticulospinal tract for strength and motor recovery: a scoping review of nonhuman and human studies. Physiol Rep 11(14):e15765 37474275 10.14814/phy2.15765 PMC10359156

[CR5] Akalu Y, Tallent J, Frazer AK, Siddique U, Rostami M, Howatson G, Walker S, Kidgell DJ (2025) Determining the cortical, corticospinal, and reticulospinal responses to metronome-paced and self-paced strength training. Eur J Appl Physiol. 10.1007/s00421-025-05939-3 40886203 10.1007/s00421-025-05939-3 PMC13013172

[CR4] Akalu Y, Tallent J, Frazer AK, Siddique U, Rostami M, Vallance P, Howatson G, Walker S, Kidgell DJ (2024) Strength-trained adults demonstrate greater corticoreticular activation versus untrained controls. Eur J Neurosci 59(9):2336–2352 38419404 10.1111/ejn.16297

[CR6] Arnold P, Bautmans I (2014) The influence of strength training on muscle activation in elderly persons: a systematic review and meta-analysis. Exp Gerontol 58:58–68 25064039 10.1016/j.exger.2014.07.012

[CR7] Atkinson E, Škarabot J, Ansdell P, Goodall S, Howatson G, Thomas K (2022) Does the reticulospinal tract mediate adaptation to resistance training in humans? J Appl Physiol 133(3):689–696 35834623 10.1152/japplphysiol.00264.2021 PMC9467470

[CR8] Baker SN (2011) The primate reticulospinal tract, hand function and functional recovery. J Physiol 589(23):5603–5612 21878519 10.1113/jphysiol.2011.215160 PMC3249036

[CR9] Baker SN, Perez MA (2017) Reticulospinal contributions to gross hand function after human spinal cord injury. J Neurosci 37(40):9778–9784 28871033 10.1523/JNEUROSCI.3368-16.2017 PMC5628413

[CR10] Barker AT, Jalinous R, Freeston IL (1985) Non-invasive magnetic stimulation of human motor cortex. Lancet 325(8437):1106–1107 10.1016/s0140-6736(85)92413-4 2860322

[CR11] Bouhrara M, Cortina LE, Rejimon AC, Khattar N, Bergeron C, Bergeron J, Melvin D, Zukley L, Spencer RG (2020) Quantitative age-dependent differences in human brainstem myelination assessed using high-resolution magnetic resonance mapping. NeuroImage 206:116307 31669302 10.1016/j.neuroimage.2019.116307 PMC6981041

[CR12] Brown P, Rothwell J, Thompson P, Britton T, Day B, Marsden C (1991) New observations on the normal auditory startle reflex in man. Brain 114(4):1891–1902 1884184 10.1093/brain/114.4.1891

[CR13] Carlsen AN, Chua R, Inglis JT, Sanderson DJ, Franks IM (2004) Prepared movements are elicited early by startle. J Mot Behav 36(3):253–264 15262622 10.3200/JMBR.36.3.253-264

[CR14] Carlsen AN, Maslovat D (2019) Startle and the StartReact effect: physiological mechanisms. J Clin Neurophysiol 36(6):452–459 31688329 10.1097/WNP.0000000000000582

[CR15] Carroll TJ, Riek S, Carson RG (2002) The sites of neural adaptation induced by resistance training in humans. J Physiol 544(2):641–652 12381833 10.1113/jphysiol.2002.024463 PMC2290590

[CR16] Caserotti P, Aagaard P, Buttrup Larsen J, Puggaard L (2008) Explosive heavy-resistance training in old and very old adults: changes in rapid muscle force, strength and power. Scan J Med Sci in Sports18(6):773–782 10.1111/j.1600-0838.2007.00732.x 18248533

[CR17] Christie A, Kamen G (2014) Cortical inhibition is reduced following short-term training in young and older adults. Age 36:749–758 23943112 10.1007/s11357-013-9577-0 PMC4039252

[CR18] Chu J, Gunraj C, Chen R (2008) Possible differences between the time courses of presynaptic and postsynaptic GABA_B_ mediated inhibition in the human motor cortex. Exp Brain Res 184:571–577 17899042 10.1007/s00221-007-1125-7

[CR19] Cirillo J, Rogasch NC, Semmler JG (2010) Hemispheric differences in use-dependent corticomotor plasticity in young and old adults. Exp Brain Res 205:57–68 20574685 10.1007/s00221-010-2332-1

[CR20] Clark B, Taylor J (2011) Age-related changes in motor cortical properties and voluntary activation of skeletal muscle. Curr Aging sci 4(3):192–199 21529329 10.2174/1874609811104030192 PMC3184350

[CR21] Colomer-Poveda D, López‐Rivera E, Hortobágyi T, Márquez G, Fernández‐Del‐Olmo M (2023) Differences in the effects of a startle stimulus on rate of force development between resistance‐trained rock climbers and untrained individuals: Evidence for reticulospinal adaptations? Scan J Med Sci Sports 33(8):1360–1372 10.1111/sms.14351 36920047

[CR22] Coombs TA, Frazer AK, Horvath DM, Pearce AJ, Howatson G, Kidgell DJ (2016a) Cross-education of wrist extensor strength is not influenced by non-dominant training in right-handers. Euro J Appl Physiol 116:1757–1769 10.1007/s00421-016-3436-5 27423912

[CR23] Coombs TA, Frazer AK, Horvath DM, Pearce AJ, Howatson G, Kidgell DJ (2016b) Cross-education of wrist extensor strength is not influenced by non-dominant training in right-handers. Euro J Appl Physiol 116(9):1757–1769 10.1007/s00421-016-3436-5 27423912

[CR24] Damron LA, Dearth DJ, Hoffman RL, Clark BC (2008) Quantification of the corticospinal silent period evoked via transcranial magnetic stimulation. J Neuro Meth 173(1):121–128 10.1016/j.jneumeth.2008.06.001 18588914

[CR25] Davidson AG, Schieber MH, Buford JA (2007) Bilateral spike-triggered average effects in arm and shoulder muscles from the monkey pontomedullary reticular formation. J Neurosci 27(30):8053–8058 17652596 10.1523/JNEUROSCI.0040-07.2007 PMC6672715

[CR26] Davis M, Gendelman DS, Tischler MD, Gendelman PM (1982) A primary acoustic startle circuit: lesion and stimulation studies. J Neurosci 2(6):791–805 7086484 10.1523/JNEUROSCI.02-06-00791.1982 PMC6564345

[CR27] Del Vecchio A, Negro F, Holobar A, Casolo A, Folland JP, Felici F, Farina D (2019) You are as fast as your motor neurons: speed of recruitment and maximal discharge of motor neurons determine the maximal rate of force development in humans. J Physiol 597(9):2445–2456 30768687 10.1113/JP277396 PMC6487919

[CR28] Doherty TJ (2003) Invited review: aging and sarcopenia. J Appl Physiol 95(4):1717–1727 12970377 10.1152/japplphysiol.00347.2003

[CR29] Evarts EV (1968) Relation of pyramidal tract activity to force exerted during voluntary movement. J Neurophysiol 31(1):14–27 4966614 10.1152/jn.1968.31.1.14

[CR30] Garson GD (2019) Multilevel Modeling: Applications in STATA^®^, IBM^®^ SPSS^®^, SAS^®^, R, & HLMTM. Sage

[CR31] Germann M, Baker SN (2021) Evidence for subcortical plasticity after paired stimulation from a wearable device. J Neurosci 41(7):1418–1428 33441436 10.1523/JNEUROSCI.1554-20.2020 PMC7896019

[CR32] Glover IS, Baker SN (2020) Cortical, corticospinal, and reticulospinal contributions to strength training. J Neurosci 40(30):5820–5832 32601242 10.1523/JNEUROSCI.1923-19.2020 PMC7380966

[CR33] Gómez-Feria J, Martín-Rodríguez JF, Mir P (2023) Corticospinal adaptations following resistance training and its relationship with strength: a systematic review and multivariate meta-analysis. Neurosci Biobehav Rev 152:105289 37353049 10.1016/j.neubiorev.2023.105289

[CR34] Gomez-Guerrero G, Avela J, Jussila I, Pihlajamäki E, Deng F-Y, Kidgell DJ, Ahtiainen JP, Walker S (2024) Cortical and spinal responses to short-term strength training and detraining in young and older adults in rectus femoris muscle. Euro J Appl Physiol 124(7):2209–2223 10.1007/s00421-024-05443-0 PMC11199260 38441691

[CR35] Gordon T, Jeanfavre M, Leff G (2024) Effects of tempo-controlled resistance training on corticospinal tract plasticity in healthy controls. A systematic review Healthcare 1325 10.3390/healthcare12131325 PMC11241463 38998859

[CR38] Hallett M (1995) Transcranial magnetic stimulation. Negative effects. Adv Neurol 67:107–113 8848963

[CR39] Hammond GR (1973) Lesions of pontine and medullary reticular formation and prestimulus inhibition of the acoustic startle reaction in rats. Physiol Behav 10(2):239–243 4575307 10.1016/0031-9384(73)90304-1

[CR40] Hedges LV, Olkin I (2014) Statistical methods for meta-analysis. Academic

[CR41] Hendy AM, Kidgell DJ (2013) Anodal tDCS applied during strength training enhances motor cortical plasticity. Med Sci Sports Exerc 45(9):1721–1729 23470308 10.1249/MSS.0b013e31828d2923

[CR42] Hermens HJ, Freriks B, Disselhorst-Klug C, Rau G (2000) Development of recommendations for SEMG sensors and sensor placement procedures. J Electromyograph Kinesiol 10(5):361–374 10.1016/s1050-6411(00)00027-4 11018445

[CR43] Herwig U, Satrapi P, Schönfeldt-Lecuona C (2003) Using the international 10–20 EEG system for positioning of transcranial magnetic stimulation. Brain Top 16:95–99 10.1023/b:brat.0000006333.93597.9d 14977202

[CR36] Häkkinen K, Alen M, Kallinen M, Newton R, Kraemer W (2000) Neuromuscular adaptation during prolonged strength training, detraining and re-strength-training in middle-aged and elderly people. Euro J Appl Physiol 83:51–62 10.1007/s004210000248 11072774

[CR37] Häkkinen K, Pakarinen A, Kraemer WJ, Häkkinen A, Valkeinen H, Alen M (2001) Selective muscle hypertrophy, changes in EMG and force, and serum hormones during strength training in older women. J Appl Physiol 91(2):569–580 11457767 10.1152/jappl.2001.91.2.569

[CR44] Hortobagyi T, Tunnel D, Moody J, Beam S, DeVita P (2001) Low-or high-intensity strength training partially restores impaired quadriceps force accuracy and steadiness in aged adults. J Gerontol Ser A: Biol Sci Med Sci 56(1):B38–B47 11193224 10.1093/gerona/56.1.b38

[CR45] Hunter SK, Pereira HM, Keenan KG (2016) The aging neuromuscular system and motor performance. J Appl Physiol 121(4):982–995 27516536 10.1152/japplphysiol.00475.2016 PMC5142309

[CR46] Jankowska E, Hammar I, Slawinska U, Maleszak K, Edgley SA (2003) Neuronal basis of crossed actions from the reticular formation on feline hindlimb motoneurons. J Neurosci 23(5):1867–1878 12629191 10.1523/JNEUROSCI.23-05-01867.2003 PMC1890022

[CR47] Jensen JL, Marstrand PC, Nielsen JB (2005) Motor skill training and strength training are associated with different plastic changes in the central nervous system. J Appl Physiol 99(4):1558–1568 15890749 10.1152/japplphysiol.01408.2004

[CR48] Kamen G, Knight CA (2004) Training-related adaptations in motor unit discharge rate in young and older adults. J Gerontol Ser A: Biol Sci Med Sci 59(12):1334–1338 15699535 10.1093/gerona/59.12.1334

[CR92] Škarabot J, Brownstein CG, Casolo A, Del Vecchio A, Ansdell P (2021) The knowns and unknowns of neural adaptations to resistance training. Eur J Appl Physiol 121:675–685 33355714 10.1007/s00421-020-04567-3 PMC7892509

[CR93] Škarabot J, Folland JP, Holobar A, Baker SN, Del Vecchio A (2022) Startling stimuli increase maximal motor unit discharge rate and rate of force development in humans. J Neurophysiol 128(3):455–469 35829632 10.1152/jn.00115.2022 PMC9423775

[CR49] Keel JC, Smith MJ, Wassermann EM (2001) A safety screening questionnaire for transcranial magnetic stimulation. Clin Neurophysiol 112(4):720 11332408 10.1016/s1388-2457(00)00518-6

[CR50] Kent-Braun JA, Ng AV (1999) Specific strength and voluntary muscle activation in young and elderly women and men. J Appl Physiol 87(1):22–29 10409554 10.1152/jappl.1999.87.1.22

[CR51] Kidgell DJ, Bonanno DR, Frazer AK, Howatson G, Pearce AJ (2017) Corticospinal responses following strength training: a systematic review and meta-analysis. Eur J Neurosci 46(11):2648–2661 28921683 10.1111/ejn.13710

[CR52] Kidgell DJ, Stokes MA, Castricum TJ, Pearce AJ (2010) Neurophysiological responses after short-term strength training of the biceps brachii muscle. J Strength Cond Res 24(11):3123–3132 20881507 10.1519/JSC.0b013e3181f56794

[CR53] Klass M, Baudry S, Duchateau J (2007) Voluntary activation during maximal contraction with advancing age: a brief review. Eur J Appl Physiol 100:543–551 16763836 10.1007/s00421-006-0205-x

[CR54] Knight C, Kamen G (2001) Adaptations in muscular activation of the knee extensor muscles with strength training in young and older adults. J Electromyograp Kinesiol 11(6):405–412 10.1016/s1050-6411(01)00023-2 11738953

[CR55] Kujirai T, Caramia M, Rothwell JC, Day B, Thompson P, Ferbert Aa, Wroe S, Asselman P, Marsden CD (1993) Corticocortical inhibition in human motor cortex. J Physiol 471(1):501–519 8120818 10.1113/jphysiol.1993.sp019912 PMC1143973

[CR56] Lambert C, Chowdhury R, FitzGerald TH, Fleming SM, Lutti A, Hutton C, Draganski B, Frackowiak R, Ashburner J (2013) Characterizing aging in the human brainstem using quantitative multimodal MRI analysis. Front Hum Neurosci 7:462 23970860 10.3389/fnhum.2013.00462 PMC3747448

[CR57] Latella C, Kidgell DJ, Pearce AJ (2012) Reduction in corticospinal inhibition in the trained and untrained limb following unilateral leg strength training. Eur J Appl Physiol 112:3097–3107 22200796 10.1007/s00421-011-2289-1

[CR58] Lee M, Gandevia SC, Carroll TJ (2009) Short-term strength training does not change cortical voluntary activation. Med Sci Sports Exerc 41(7):1452–1460 19516155 10.1249/MSS.0b013e3181998837

[CR59] Leung M, Rantalainen T, Teo W-P, Kidgell D (2017) The corticospinal responses of metronome-paced, but not self-paced strength training are similar to motor skill training. Eur J Appl Physiol 117:2479–2492 29018949 10.1007/s00421-017-3736-4

[CR60] Maitland S, Baker SN (2021) Ipsilateral motor evoked potentials as a measure of the reticulospinal tract in age-related strength changes. Front Aging Neurosci 13:612352 33746734 10.3389/fnagi.2021.612352 PMC7966512

[CR61] Manini TM, Visser M, Won-Park S, Patel KV, Strotmeyer ES, Chen H, Goodpaster B, De Rekeneire N, Newman AB, Simonsick EM (2007) Knee extension strength cutpoints for maintaining mobility. J Am Geriat Soc 55(3):451–457 17341251 10.1111/j.1532-5415.2007.01087.x

[CR62] Marques DL, Neiva HP, Marinho DA, Marques MC (2023) Manipulating the resistance training volume in middle-aged and older adults: a systematic review with meta-analysis of the effects on muscle strength and size, muscle quality, and functional capacity. Sports Med 53(2):503–518 36307745 10.1007/s40279-022-01769-x

[CR63] Mason J, Frazer A, Horvath DM, Pearce AJ, Avela J, Howatson G, Kidgell D (2017) Adaptations in corticospinal excitability and inhibition are not spatially confined to the agonist muscle following strength training. Eur J Appl Physiol 117(7):1359–1371 28455814 10.1007/s00421-017-3624-y

[CR64] Mason J, Frazer AK, Avela J, Pearce AJ, Howatson G, Kidgell DJ (2020) Tracking the corticospinal responses to strength training. Eur J Appl Physiol 120:783–798 32060740 10.1007/s00421-020-04316-6

[CR65] McDonnell MN, Orekhov Y, Ziemann U (2007) Suppression of LTP-like plasticity in human motor cortex by the GABA B receptor agonist baclofen. Exp Brain Res 180:181–186 17351767 10.1007/s00221-006-0849-0

[CR66] McEntee WJ, Crook TH (1993) Glutamate: its role in learning, memory, and the aging brain. Psychopharma 111(4):391–401 10.1007/BF02253527 7870979

[CR69] Müller-Dahlhaus F, Ziemann U (2015) Metaplasticity in human cortex. Neurosci 21(2):185–202 10.1177/1073858414526645 24620008

[CR67] Mooney RA, Bastian AJ, Celnik PA (2023) Mapping subcortical motor pathways in humans with startle-conditioned TMS. Brain Stim 16(5):1232–1239 10.1016/j.brs.2023.08.010 PMC11724745 37595834

[CR68] Moritani T, DeVries HA (1979) Neural factors versus hypertrophy in the time course of muscle strength gain. Am J Phys Med Rehabil 58(3):115–130 453338

[CR70] Nonnekes J, Oude Nijhuis LB, de Niet M, de Bot ST, Pasman JW, van de Warrenburg BP, Bloem BR, Weerdesteyn V, Geurts AC (2014) StartReact restores reaction time in HSP: evidence for subcortical release of a motor program. J Neurosci 34(1):275–281 24381288 10.1523/JNEUROSCI.2948-13.2014 PMC6608175

[CR71] Oldfield R (1971) Edinburgh handedness inventory. J Abnorm Psych 9(1):97–113 10.1016/0028-3932(71)90067-4 5146491

[CR72] Oliviero A, Profice P, Tonali P, Pilato F, Saturno E, Dileone M, Ranieri F, Di Lazzaro V (2006) Effects of aging on motor cortex excitability. Neurosci Res 55(1):74–77 16584795 10.1016/j.neures.2006.02.002

[CR73] Opie GM, Semmler JG (2014) Age-related differences in short- and long-interval intracortical inhibition in a human hand muscle. Brain Stim 7(5):665–672 10.1016/j.brs.2014.06.014 25088463

[CR74] Palmieri RM, Ingersoll CD, Hoffman MA (2004) The Hoffmann reflex: methodologic considerations and applications for use in sports medicine and athletic training research. J Athl Train 39(3):268 16558683 PMC522151

[CR75] Patten C, Kamen G, Rowland DM (2001) Adaptations in maximal motor unit discharge rate to strength training in young and older adults. Muscle Nerve 24(4):542–550 11268027 10.1002/mus.1038

[CR76] Pearce A, Hendy A, Bowen W, Kidgell D (2013) Corticospinal adaptations and strength maintenance in the immobilized arm following 3 weeks unilateral strength training. Scand J Med Sci Sports 23(6):740–748 22429184 10.1111/j.1600-0838.2012.01453.x

[CR77] Peinemann A, Lehner C, Conrad B, Siebner HR (2001) Age-related decrease in paired-pulse intracortical inhibition in the human primary motor cortex. Neuroscice lett 313(1–2):33–36 10.1016/s0304-3940(01)02239-x 11684333

[CR78] Perez MA, Cohen LG (2009) Scaling of motor cortical excitability during unimanual force generation. Cortex 45(9):1065–1071 19243741 10.1016/j.cortex.2008.12.006

[CR79] Peterson B, Maunz R, Pitts N, Mackel R (1975) Patterns of projection and branching of reticulospinal neurons. Exp Brain Res 23(4):333–351 1183508 10.1007/BF00238019

[CR80] Pitcher JB, Ogston KM, Miles TS (2003) Age and sex differences in human motor cortex input–output characteristics. J Physiol 546(2):605–613 12527746 10.1113/jphysiol.2002.029454 PMC2342521

[CR109] R. A. Mooney and P. A. Celnik. (2025). Eff ector-dependent decline in strength and subcortical motor excitabilitywith aging. Neurobiology of Aging 2025 Vol. 147 Pages 98-104. 10.1016/j.neurobiolaging.2024.12.008 39733761

[CR81] Rantanen T, Guralnik JM, Foley D, Masaki K, Leveille S, Curb JD, White L (1999) Midlife hand grip strength as a predictor of old age disability. JAMA 281(6):558–560 10022113 10.1001/jama.281.6.558

[CR82] Riddle CN, Edgley SA, Baker SN (2009) Direct and indirect connections with upper limb motoneurons from the primate reticulospinal tract. J Neurosci 29(15):4993–4999 19369568 10.1523/JNEUROSCI.3720-08.2009 PMC2690979

[CR83] Rogasch NC, Dartnall TJ, Cirillo J, Nordstrom MA, Semmler JG (2009) Corticomotor plasticity and learning of a ballistic thumb training task are diminished in older adults. J Appl Physiol 107(6):1874–1883 19833810 10.1152/japplphysiol.00443.2009

[CR84] Rossini PM, Burke D, Chen R, Cohen LG, Daskalakis Z, Di Iorio R, Di Lazzaro V, Ferreri F, Fitzgerald P, George MS (2015) Non-invasive electrical and magnetic stimulation of the brain, spinal cord, roots and peripheral nerves: Basic principles and procedures for routine clinical and research application. An updated report from an IFCN Committee. Clin Neurophysiol 126(6):1071–1107 25797650 10.1016/j.clinph.2015.02.001 PMC6350257

[CR85] Rothwell J (2006) The startle reflex, voluntary movement, and the reticulospinal tract. Suppl Clin Neurophysiol 58:223–231 16623334 10.1016/s1567-424x(09)70071-6

[CR86] Sale DG (1988) Neural adaptation to resistance training. Med Sci Sports Exerc 20(5):S135–S145 3057313 10.1249/00005768-198810001-00009

[CR87] Sale MV, Semmler JG (2005) Age-related differences in corticospinal control during functional isometric contractions in left and right hands. J Appl Physiol 99(4):1483–1493 15947031 10.1152/japplphysiol.00371.2005

[CR88] Sangari S, Perez MA (2020) Distinct corticospinal and reticulospinal contributions to voluntary control of elbow flexor and extensor muscles in humans with tetraplegia. J Neurosci 40(46):8831–8841 32883710 10.1523/JNEUROSCI.1107-20.2020 PMC7659455

[CR89] Siddique U, Frazer AK, Avela J, Walker S, Ahtiainen JP, Howatson G, Tallent J, Kidgell DJ (2022) Determining the cortical, spinal and muscular adaptations to strength-training in older adults: a systematic review and meta-analysis. Ageing Res Rev 82:101746 36223874 10.1016/j.arr.2022.101746

[CR90] Siddique U, Frazer AK, Avela J, Walker S, Ahtiainen JP, Tanel M, Uribe S, Akalu Y, Rostami M, Tallent J (2024) Differential modulation of corticomotor excitability in older compared to young adults following a single bout of strength-exercise. Arch Gerontol Geriat 122:105384 10.1016/j.archger.2024.105384 38394740

[CR91] Siddique U, Rahman S, Frazer AK, Pearce AJ, Howatson G, Kidgell DJ (2020) Determining the sites of neural adaptations to resistance training: a systematic review and meta-analysis. Sports Med 50:1107–1128 31993949 10.1007/s40279-020-01258-z

[CR94] Stinear CM, Coxon JP, Byblow WD (2009) Primary motor cortex and movement prevention: where Stop meets Go. Neurosci Biobehavl Rev 33(5):662–673 10.1016/j.neubiorev.2008.08.013 18789963

[CR95] Tapia JA, Tohyama T, Poll A, Baker SN (2022) The existence of the StartReact effect implies reticulospinal, not corticospinal, inputs dominate drive to motoneurons during voluntary movement. J Neurosci 42(40):7634–7647 36658461 10.1523/JNEUROSCI.2473-21.2022 PMC9546468

[CR96] Todd G, Kimber TE, Ridding MC, Semmler JG (2010) Reduced motor cortex plasticity following inhibitory rTMS in older adults. Clin Neurophysiol 121(3):441–447 20071228 10.1016/j.clinph.2009.11.089

[CR108] Ummatul Siddique AK, Frazer J, Tallent O, Hayman J, Andrushko, Juha P, Ahtiainen JA, Akalu Y (2025) Mohamad Rostami, Sergio Uribe, Simon Walker, Dawson J Kidgell. Acute corticospinal and reticulospinal responses to strength training in ageing. Neurobiology of Aging, 153, 49-62 10.1016/j.neurobiolaging.2025.06.007 40578297

[CR97] Unhjem R, Lundestad R, Fimland MS, Mosti MP, Wang E (2015) Strength training-induced responses in older adults: attenuation of descending neural drive with age. Age 37:1–13 25940749 10.1007/s11357-015-9784-y PMC4418975

[CR99] Valls-Solé J, Rothwell JC, Goulart F, Cossu G, Muñoz E (1999) Patterned ballistic movements triggered by a startle in healthy humans. J Physiol 516(3):931–938 10200438 10.1111/j.1469-7793.1999.0931u.x PMC2269293

[CR98] Valls-Solé J, Solé A, Valldeoriola F, Muñoz E, Gonzalez L, Tolosa E (1995) Reaction time and acoustic startle in normal human subjects. Neurosci lett 195(2):97–100 7478277 10.1016/0304-3940(94)11790-p

[CR100] Van Cutsem M, Duchateau J, Hainaut K (1998) Changes in single motor unit behaviour contribute to the increase in contraction speed after dynamic training in humans. J Physiol 513(1):295–305 9782179 10.1111/j.1469-7793.1998.295by.x PMC2231276

[CR101] Visser M, Deeg DJ, Lips P, Harris TB, Bouter LM (2000) Skeletal muscle mass and muscle strength in relation to lower-extremity performance in older men and women. J Am Geriat Soc 48(4):381–386 10798463 10.1111/j.1532-5415.2000.tb04694.x

[CR102] Walker S, Häkkinen K (2014) Similar increases in strength after short-term resistance training due to different neuromuscular adaptations in young and older men. J Strength Cond Res 28(11):3041–3048 25051001 10.1519/JSC.0000000000000381

[CR103] Walker S, Peltonen H, Avela J, Häkkinen K (2013) Neuromuscular fatigue in young and older men using constant or variable resistance. Eur JAppl Physiol 113:1069–1079 23079866 10.1007/s00421-012-2526-2

[CR104] Walker S, Tanel M, Vekki S, Kidgell DJ, Baker SN (2024) The effects of the StartReact on reaction time, rate of force development, and muscle activity in biceps brachii. Scand J Med Sci Sports 34(9):e14733 10.1111/sms.14733 39308053

[CR105] Weier A, Pearce A, Kidgell D (2012) Strength training reduces intracortical inhibition. Acta Physiol 206(2):109–119 10.1111/j.1748-1716.2012.02454.x 22642686

[CR106] Werhahn KJ, Kunesch E, Noachtar S, Benecke R, Classen J (1999) Differential effects on motorcortical inhibition induced by blockade of GABA uptake in humans. J Physiol 517(2):591–597 10332104 10.1111/j.1469-7793.1999.0591t.x PMC2269337

[CR107] Wilkinson RD, Mazzo MR, Feeney DF (2023) Rethinking the statistical analysis of neuromechanical data. Exerc Sport Sci rEV 51(1):43–50 36206407 10.1249/JES.0000000000000308

