# Supplementary material for: Age-related differences in corticospinal and reticulospinal adaptations to short-term strength training
Source: Eur J Appl Physiol. 2026 Apr 10;126(7):4095–112. doi: 10.1007/s00421-026-06222-9 (PMC13380581; doi:10.1007/s00421-026-06222-9)
Supplement: Supplementary file 1 — Supplementary Material 1 [file 421_2026_6222_MOESM1_ESM.docx]

**Table 1.** Mean (95%CI) values for demographic and strength variables at baseline and post the two-week training period.

|  | **Older Adults** | | **Young Adults** | |
| --- | --- | --- | --- | --- |
|  | **Pre** | **Post** | **Pre** | **Post** |
| **Age(years)** | 66  (63-70) | | 26  (22-29) | |
| **Sex (Male: Female)** | 5:7 | | 6:6 | |
| **Body mass (kg)** | 77  (67-86) | | 69  (62-76) | |
| **Height (cm)** | 165.7  (160.2-171.2) | | 170.3  (163.7-176.8) | |
| **1RM (kg)** | 10.3  (8.3-12.3) | 12.0  (10.0-14.1) | 10.5  (7.7-13.3) | 12.4  (9.4-15.5) |
| **1-RM rmsEMG(%M_MAX_)** | **4.2^##^**  **(2.6-5.7)** | 4.9  (3.4-6.4) | **7.1^##^**  **(4.4-9.8)** | 5.4  (3.6-7.3) |
| **MVF (N)** | 82.7  (64.5-100.8) | **96.1^^^^**  **(73.6-118.5)** | 95.9  (71.6-120.1) | 102.5  (77.5-127.6) |
| **MVF rmsEMG (%M_MAX_)** | 5.3  (3.6-7.1) | 5.6  (3.4-7.8) | 7.5  (4.5-10.5) | 6.4  (5.2-7.6) |

*1-RM* one repetition maximum, *rmsEMG* root-mean-square electromyography, *MVF* maximum voluntary force, **^##^**p<0.01 denotes a significant baseline difference in 1-RM rmsEMG (%M_MAX_) between older and younger adults. ^^ p<0.001 denotes a significant difference in MVF (N) from pre-post in older adults.

**Table 2:** Mean (95%CI) values for neurophysiological measures at baseline, post two-week training intervention in older and younger adults.

|  | | **Older Adults** | | | **Young Adults** | | |
| --- | --- | --- | --- | --- | --- | --- | --- |
|  | | **Pre** | **Post** | **Effect Size**  **(g)** | **Pre** | **Post** | **Effect Size**  **(g)** |
| **AMT SI (%)** | | 49  (43-54) | 44  (39-49) | ***0.51*** | 45  (40-49) | 41  (38-45) | ***0.47*** |
| **Corticospinal Excitability CSE (%M_MAX_)** | **100%AMT** | 5.66  (0.36-10.96) | 5.50  (0.20-10.80) | ***0.06*** | 5.44  (0.08-10.95) | 4.69  (0.83-10.21) | ***0.40*** |
|  | **130%AMT** | 19.45  (14.15-24.75) | 19.20  13.90-24.50) | ***0.16*** | 25.83  (20.32-31.35) | **14.28^***^**  **(8.77-19.80)** | ***1.18*** |
|  | **150%AMT** | 29.19  (23.89-34.49) | 30.44  (31.21-42.24) | ***0.02*** | 36.72  (25.14-35.74) | **25.95^***^**  **(20.43-31.46)** | ***0.71*** |
|  | **170%AMT** | 36.38  (31.08-41.68) | 36.72  (36.93-47.96) | ***0.06*** | 42.44  (31.42-42.02) | **32.64^***^**  **(27.13-38.16)** | ***0.50*** |
| **Cortical Silent Period**  **cSP (ms)** | **130%AMT** | 109.79  (93.54-126.03) | **95.99^†^**  **(79.74-112.26)** | ***0.29*** | 100.47  (83.53-117.42) | **100.65^†^**  **(83.66-117.63)** | ***0.08*** |
|  | **150%AMT** | 132.77  116.53-149.02) | **119.06^†††^**  **(102.79-135.32)** | ***0.39*** | 132.30  (115.37-149.23) | **119.401^†††^**  **(102.48-136.33)** | ***0.43*** |
|  | **170%AMT** | 156.42  (140.17-172.66) | **135.78^†††^**  **(119.54-152.03)** | ***0.49*** | 156.56  (139.65-173.46) | **139.74^†††^**  **(122.79-156.69)** | ***0.69*** |
| **SICI (% Test Response)** | | **47.68^#^**  **(39.85-55.52)** | 52.67  (44.97-60.37) | ***0.31*** | **65.64^#^**  **(57.56-73.72)** | 72.07  (64.29-79.85) | ***0.23*** |
| **ICF (% Test Response)** | | 142.27  (122.08-162.46) | 134.16  (115.14-153.18) | ***0.19*** | 141.88  (122.76-160.99) | 143.63  (124.49-162.77) | ***0.08*** |
| **CAR (%)** | | 96.0  (94.90-97.10) | **98.70^^^^^**  **(97.6--99.80)** | ***0.98*** | 98.40  (97.20-99.60) | 98.90  (97.70-100.00) | ***0.60*** |

*AMT* active motor threshold*, SI* stimulator input*, MEP* motor evoked potential, *M_MAX_* maximum compound action potential, *CSE* corticospinal excitability*, cSP* cortical silent period, *SICI* short-interval intracortical inhibition, *ICF* intracortical facilitation. *CAR* Central Activation Ratio. Effect Size (hedge’s g) represents within group changes across time. ***p<0.001 indicates a significant decrease in CSE from pre-in young adults whereas **^^^^^**p<0.001 indicate a significant increase in CAR post training in older adults. **^#^**p<0.05 denotes significant difference in SICI between older and young adults at baseline. **^†^**p<0.05 and ^†††^p<0.001 indicate a significant main effect of time (collapsed across groups) for cSP at 130, 150, and 170 % AMT.

|  | | **Older Adults** | | | **Young Adults** | | |
| --- | --- | --- | --- | --- | --- | --- | --- |
|  | | **Pre** | **Post** | **Effect Size**  **(g)** | **Pre** | **Post** | **Effect Size**  **(g)** |
| **Reaction Time**  **(ms)** | **VRT** | 187.34  (168.45-206.23) | 178.812  (159.83-197.79) | ***0.32*** | 183.71  (164.05203.37) | 175.95  (156.29-195.61) | ***0.19*** |
|  | **VART** | 125.89  (107.00-144.78) | 114.59  (95.60-133.57) | ***0.43*** | 120.14  (100.48-139.81) | 111.77  (92.10-131.43) | ***0.17*** |
|  | **VSRT** | 97.85  (78.96-116.74) | 75.73  (56.7494.71) | ***1.11*** | 100.19  (80.54-119.86) | 89.05  (69.39-108.71) | ***0.23*** |
|  | **SR*_Effect_*** | 28.05  (19.34-36.75) | **38.86^##^**  **(30.15-47.56)** | ***0.66*** | 19.95  (10.89-29.00) | 22.72  (13.66-31.77) | ***0.16*** |
| **RFD(N/s)**  **0-50ms** | **VRT** | 466.15  (338.89-593.41) | 607.80  (480.55-735.06) | ***0.56*** | 573.39  (435.04-711.73) | 662.66  (524.32-801.00) | ***0.29*** |
|  | **VART** | 489.16  (361.89-616.41) | 633.88  (506.62-761.14) | ***0.53*** | 612.23  (473.88-750.57) | 667.29  (528.95-805.64) | ***0.16*** |
|  | **VSRT** | 529.59  (402.33-656.85) | **791.95^##^**  **(664**.**69-919.20)** | ***0.86*** | 628.05  (489.70-766.39) | **848.24^##^**  **(709.89-986.58)** | ***0.55*** |
| **RFD(N/s)**  **50-100ms** | **VRT** | 229.49  (102.23-356.75) | 263.18  (135.93-390.44) | ***0.24*** | 268.76  (130.42-407.11) | 262.39  (124.05-400.73) | ***0.04*** |
|  | **VART** | 247.24  (119.99-374.50) | 263.93  (136.68-391.19) | ***0.11*** | 252.47  (114.12-390.81) | 253.05  (114.71-391.39) | ***0.00*** |
|  | **VSRT** | 227.32  (100.07-354.58) | 241.32  (114.06-368.58) | ***0.10*** | 259.79  (121.46-398.14) | 258.53  (120.19-396.87) | ***0.01*** |

**Table 3.** Mean (95%CI) values for reaction time and rate of force development across the training intervention in older and younger adults.

*SR_Effect_* startreact effect*, RFD* rate of force development, *VRT* visual reaction time, *VART* visual-acoustic reaction time, *VSRT* visual startle reaction time. **^##^**p<0.01 indicate a significant difference at post.
